# Circulating Small Noncoding RNAs Have Specific Expression Patterns in Plasma and Extracellular Vesicles in Myelodysplastic Syndromes and Are Predictive of Patient Outcome

**DOI:** 10.3390/cells9040794

**Published:** 2020-03-26

**Authors:** Andrea Hrustincova, Zdenek Krejcik, David Kundrat, Katarina Szikszai, Monika Belickova, Pavla Pecherkova, Jiri Klema, Jitka Vesela, Monika Hruba, Jaroslav Cermak, Tereza Hrdinova, Matyas Krijt, Jan Valka, Anna Jonasova, Michaela Dostalova Merkerova

**Affiliations:** 1 Institute of Hematology and Blood Transfusion, U Nemocnice 1, 128 20 Prague, Czech Republic; andrea.mrhalkova@uhkt.cz (A.H.); zdenek.krejcik@uhkt.cz (Z.K.); david.kundrat@uhkt.cz (D.K.); katarina.szikszai@uhkt.cz (K.S.); monika.belickova@uhkt.cz (M.B.); pavla.pecherkova@uhkt.cz (P.P.); jitka.vesela@uhkt.cz (J.V.); monika.hruba@uhkt.cz (M.H.); jaroslav.cermak@uhkt.cz (J.C.); tereza.kabickova@uhkt.cz (T.H.); matyas.krijt@uhkt.cz (M.K.); jan.valka@uhkt.cz (J.V.); 2 Faculty of Science, Charles University, Albertov 2038, 128 00 Prague, Czech Republic; 3 Czech Technical University, Karlovo namesti 13, 121 35 Prague, Czech Republic; klema@fel.cvut.cz; 4 First Faculty of Medicine, Charles University, Kateřinská 1660/32, 121 08 Prague, Czech Republic; 5 General University Hospital, U Nemocnice 499/2, 128 08 Prague, Czech Republic; atjonas@hotmail.com

**Keywords:** myelodysplastic syndromes, circulating small noncoding RNAs, extracellular vesicles, biomarkers

## Abstract

Myelodysplastic syndromes (MDS) are hematopoietic stem cell disorders with large heterogeneity at the clinical and molecular levels. As diagnostic procedures shift from bone marrow biopsies towards less invasive techniques, circulating small noncoding RNAs (sncRNAs) have become of particular interest as potential novel noninvasive biomarkers of the disease. We aimed to characterize the expression profiles of circulating sncRNAs of MDS patients and to search for specific RNAs applicable as potential biomarkers. We performed small RNA-seq in paired samples of total plasma and plasma-derived extracellular vesicles (EVs) obtained from 42 patients and 17 healthy controls and analyzed the data with respect to the stage of the disease, patient survival, response to azacitidine, mutational status, and RNA editing. Significantly higher amounts of RNA material and a striking imbalance in RNA content between plasma and EVs (more than 400 significantly deregulated sncRNAs) were found in MDS patients compared to healthy controls. Moreover, the RNA content of EV cargo was more homogeneous than that of total plasma, and different RNAs were deregulated in these two types of material. Differential expression analyses identified that many hematopoiesis-related miRNAs (e.g., miR-34a, miR-125a, and miR-150) were significantly increased in MDS and that miRNAs clustered on 14q32 were specifically increased in early MDS. Only low numbers of circulating sncRNAs were significantly associated with somatic mutations in the *SF3B1* or *DNMT3A* genes. Survival analysis defined a signature of four sncRNAs (miR-1237-3p, U33, hsa_piR_019420, and miR-548av-5p measured in EVs) as the most significantly associated with overall survival (HR = 5.866, *p* < 0.001). In total plasma, we identified five circulating miRNAs (miR-423-5p, miR-126-3p, miR-151a-3p, miR-125a-5p, and miR-199a-3p) whose combined expression levels could predict the response to azacitidine treatment. In conclusion, our data demonstrate that circulating sncRNAs show specific patterns in MDS and that their expression changes during disease progression, providing a rationale for the potential clinical usefulness of circulating sncRNAs in MDS prognosis. However, monitoring sncRNA levels in total plasma or in the EV fraction does not reflect one another, instead, they seem to represent distinctive snapshots of the disease and the data should be interpreted circumspectly with respect to the type of material analyzed.

## 1. Introduction

Myelodysplastic syndromes (MDS) are a heterogeneous group of hematopoietic stem cell disorders characterized by bone marrow (BM) dysplasia, ineffective hematopoiesis, cytopenias in peripheral blood (PB) and increased tendency towards transformation to acute myeloid leukemia (AML). MDS subtype classification is based on determination of the blast cell percentage in bone marrow, cytogenetic detection of del(5q), PB cytopenias and the presence of ringed sideroblasts [1]. To assess patient prognosis, the Revised International Prognostic Scoring System (IPSS-R) [2] to evaluate clinicopathological characteristics was developed. The main treatment option for MDS patients with higher risk disease who are not eligible for hematopoietic stem cell transplantation (HSCT) is the hypomethylating agent azacytidine (AZA). However, considering the high heterogeneity of MDS and that only a portion of patients respond to AZA (40–50%), there is still a need to discover new biomarkers that would improve the existing prognostic system or predict patient responses to medication.

MiRNAs, a class of small noncoding RNA (sncRNA) molecules, are important regulators of many essential cellular processes. It has been discovered that miRNAs are released into various body fluids in complexes with lipoproteins [3] or proteins [4,5] or encapsulated in extracellular vesicles (EVs) [6,7]. The latter enable a transfer of their molecular cargo to recipient cells via transfer through the blood circulation, which plays an important role in long distance cell-to-cell communication [8,9,10]. These blood-based miRNAs are referred to as “circulating miRNAs”. The secretion of miRNAs seems to be a controlled, active, and specific process as they are selectively included in EVs [6,11,12]. These observations indicate that circulating miRNAs may reflect physiological and pathological processes occurring in different cells and tissues, and so might be valuable blood-based biomarkers of various diseases. Additionally, blood plasma and EVs are of special interest because they can be obtained noninvasively, offering a novel feasible alternative to routine invasive BM biopsies that can be demanding, especially for elderly patients with many comorbidities.

MiRNAs represent the most explored sncRNA species in humans [13]. However, there are other types of sncRNAs, such as piwi-interacting RNAs (piRNAs), transfer RNA (tRNA), small nuclear RNAs (snRNAs), and small nucleolar RNAs (snoRNAs). All of these have been found in plasma and EVs along with miRNAs and have become of particular interest to the field. However, the origin and role of other types of circulating sncRNAs in health and disease are still unknown and remain to be elucidated.

To date, only a few studies have investigated circulating sncRNAs in MDS. Levels of let-7a, miR-16 [14] and miR-21 [15] were analyzed in blood plasma and serum, and the data showed that these miRNAs could serve as prognostic biomarkers of MDS. Zuo et al. [16] were the first to investigate the global profile of circulating miRNAs in MDS plasma, and they identified a 7-miRNA signature as an independent predictor of survival in MDS patients with normal karyotypes. We investigated the circulating miRNA profile in MDS plasma and found that the levels of several miRNAs (miR-27a-3p, miR-150-5p, miR-199a-5p, miR-223-3p, and miR-451a) were reduced in higher-risk MDS compared to lower-risk disease and that the levels of miR-451a and miR-223-3p were predictive of patient outcome [17]. Giudice et al. [18] examined the possible diagnostic and prognostic potential of plasma exosomal miRNAs and found 21 miRNAs that had a strong association with MDS. Finally, Enjeti et al. [19] studied the sncRNA content in EVs of MDS patients and revealed that their cargo was approximately twice as high as that in EVs of the healthy controls.

In this study, we performed small RNA-seq analysis to investigate circulating sncRNA profiles in MDS to better understand MDS pathogenesis and to search for specific RNAs as potential disease biomarkers. This is the first MDS study that analyzed paired samples from total plasma and the EV fraction and compared their sncRNA profiles with the aim of defining whether the sncRNA contents of plasma and EVs reflect each other or not and which of these two materials would be a better source of relevant RNA biomarkers for MDS.

## 2. Results

### 2.1. Patient Characteristics

The small RNA-seq study was conducted on a cohort of 59 individuals, which included 31 MDS patients, 11 AML-MRC patients, and 17 controls. The samples were obtained from yet untreated patients. The time from diagnosis ranged from 0 months (i.e., diagnostic samples) to 5 years from the initial assessment of the diagnosis, with the majority of samples obtained within 1 year from the diagnosis (81%). Based on the WHO 2016 classification criteria [1], the diagnoses of MDS patients were as follows: 5 MDS patients with multilineage dysplasia (MDS-MLD), 5 MDS patients with ring sideroblasts (MDS-RS), 3 MDS patients with isolated del(5q), 5 MDS patients with excess blasts 1 (MDS-EB1), and 13 MDS patients with excess blasts 2 (MDS-EB2). The detailed clinical and laboratory characteristics of the cohort that include diagnostic classification, IPSS-R category, bone marrow features, blood counts, cytogenetics, mutational screening results, and follow-up information, are summarized in SI 1.

The results from routine mutational screening (for 54 genes associated with myeloid malignancies) were available for 18 of the 31 MDS patients (58%). Of them, 83% bore at least one somatic mutation with 1.8 mutational events per patient on average (range 1–5). The most frequently mutated genes in the cohort were *SF3B1* (5 patients, 28%, variant allele frequency (VAF) ranging from 26% to 50%) and *DNMT3A* (5 patients, 28%, VAF ranging from 26% to 47%) (SI 2). These two most commonly mutated genes were selected for further investigation.

In the follow-up period, 24 patients received AZA therapy. The mean time from sample collection to AZA initiation was 1 month (0–7 months). The mean number of administered AZA cycles was 9 (2–56 cycles), the mean time to the best response in the responder cohort was 4.5 months (3–6 months), and the mean duration of the response was 15 months (6–56 months). In this AZA cohort, 9 patients were considered responders (i.e., they achieved complete remission, partial remission, marrow complete remission, or hematological improvement), 6 patients had stable disease, and 9 patients progressed after AZA initiation. The overall response rate (ORR) that included rates for all responders within the cohort was 37.5%.

Furthermore, we analyzed the survival of MDS patients stratified according to clinical variables. We found that diagnosis, IPSS-R category, IPSS-R-based karyotype, bone marrow blasts, hemoglobin level, and platelet count were significantly associated with overall survival (OS) (SI 3).

Additionally, an independent validation cohort comprising 36 MDS patients, 7 AML-MRC patients, and 12 controls was analyzed by ddPCR. The characteristics of the validation cohort are summarized in SI 4.

### 2.2. Characterization of Extracellular Vesicles in MDS Plasma

To characterize the EVs in MDS plasma, we analyzed several total plasma samples by nanoparticle tracking analysis (NTA). We observed that neither particle counts nor their cumulative volumes were associated with the disease. However, we observed a fraction of particles with larger sizes specifically in the plasma of higher risk MDS patients (SI 5A and SI 5B). More importantly, we obtained significantly higher amounts of RNA material from patient samples compared to healthy donors when isolated from total plasma samples (44.7 ± 3.2 ng/mL of plasma in healthy controls vs. 92.9 ± 8.8 ng/mL in MDS samples, *p* = 0.01) as well as from EV fractions (7.3 ± 0.5 ng/mL of plasma in healthy controls vs. 17.5 ± 1.3 ng/mL in MDS samples, *p* = 0.0005) (SI 5C).

After separation, we reanalyzed the isolated EVs by transmission electron microscopy (TEM), NTA, and Western blotting. TEM imaging showed that the majority of EVs ranged in size from 50 nm to 100 nm, suggesting sufficient exosome enrichment in the samples (SI 6A). Based on the NTA measurements, we determined that the obtained EVs had a mode size of 96 nm (the size that has the highest number of recurrences in the sample), with particle sizes ranging from 87 nm to 180 nm (10th to 90th percentile) (SI 6B). By Western blotting, we confirmed the presence of exosomes. Common exosome markers CD9 and CD81 were detected in MDS and control EV samples, whereas cell organelle (endoplasmic reticulum) marker calnexin tested as a negative control was not detected (SI 6C). Based on these data, we conclude that although enriched in exosomes, our precipitated EV fractions contain a heterogeneous mixture of exosomes and, to a lesser extent, microvesicles. This is consistent with outputs from other protocols that include high-speed ultracentrifugation (e.g., [20]).

### 2.3. General Overview of Circulating sncRNAs of MDS Patients

To systematically characterize the sncRNAs circulating in MDS plasma, we performed small RNA-seq analysis in paired samples (118 samples from 59 individuals) of total plasma and plasma-derived EVs. On average, we captured 8.3 millions (M) of total reads per sample (4.0–18.0 M reads) and after filtering, 5.0 M reads on average per sample (2.6–12.6 M reads) were retained in the analysis. In the filtering process, we removed all reads that had low quality scores, defective or missing adapters and/or barcodes, and were shorter than 16 nucleotides. The data have been deposited in the SRA (Sequence Read Archive) database under accession no. PRJNA574254.

The retained sequences were annotated and assigned to several categories of transcripts. The most abundant category of transcripts were miRNAs (51.0% of reads on average), followed by rRNAs (4.1%), piRNAs (0.7%), tRNAs (0.4%), and mRNAs (0.4%). Of the retained reads, 42.2% were uncharacterized (of these, 8.5% were mappable and 33.7% were not mappable to the human genome). In total, we identified 2543 miRNAs, 141 piRNAs and 364 tRNAs with at least one read in one sample.

Because a large proportion of the mapped reads were uncharacterized, we predicted potential de novo miRNAs among these sequences. Using the miRdeep2 tool, we identified 7667 de novo miRNAs in total. On average, we found 168 and 146 de novo miRNAs per sample of total plasma and EVs, respectively. These de novo identified miRNAs were included in subsequent differential analyses. 

Furthermore, we addressed the question of the features and origin of the uncharacterized unmapped reads. Interestingly, their proportion increased with read length. While almost all of the reads with lengths of 20–22 bp mapped to the human genome, the unmapped reads were longer (>31 bp, SI 7). A high percentage of unmapped sequences was annotated as 16S/18S rRNA molecules of nonhuman origin (approx. 20%), suggesting that numerous RNAs originating from other species are present in human plasma. The majority of these reads were mappable to *Proteobacteria*, *Ascomycota*, *Streptophyta*, and *Chlorophyta*. No apparent profile specific to MDS patients either in total plasma or in EVs was found (SI 8). However, a deeper analysis of these nonhuman sequences with respect to differential representation of individual taxa has not been done as is beyond the scope of this study.

### 2.4. Comparison of sncRNAs Circulating in Total Plasma vs. Encapsulated in EVs

**Figure 1:**
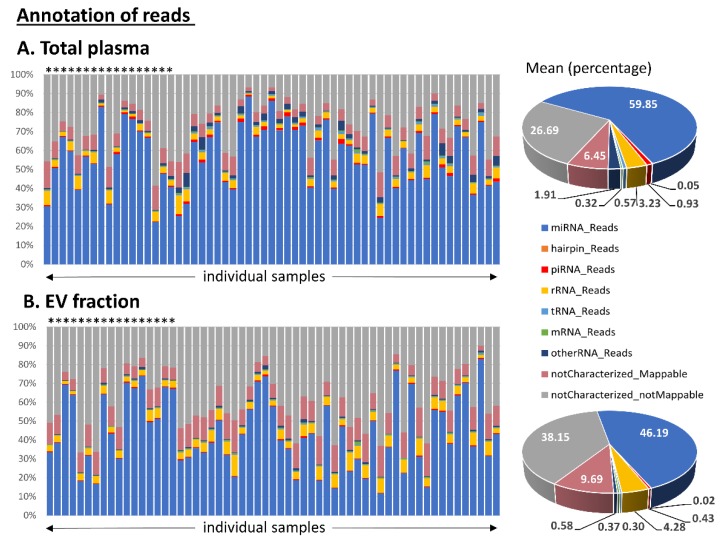
Annotation of small RNA-seq data outputs of (**A**) total plasma samples and (**B**) samples from corresponding extracellular vesicles (EVs). The read percentage was calculated from the total number of annotated read counts after quality control filtering. Healthy control samples are marked with stars above the bars. The pie charts show the mean distribution of reads in the two types of material.

We focused on human sncRNAs and observed that the total plasma samples had a substantially higher proportion of miRNA reads compared to the EV samples. In contrast, EV samples had higher numbers of uncharacterized reads. The detailed distribution of the reads in the annotation categories is shown in Figure 1.

**Figure 2:**
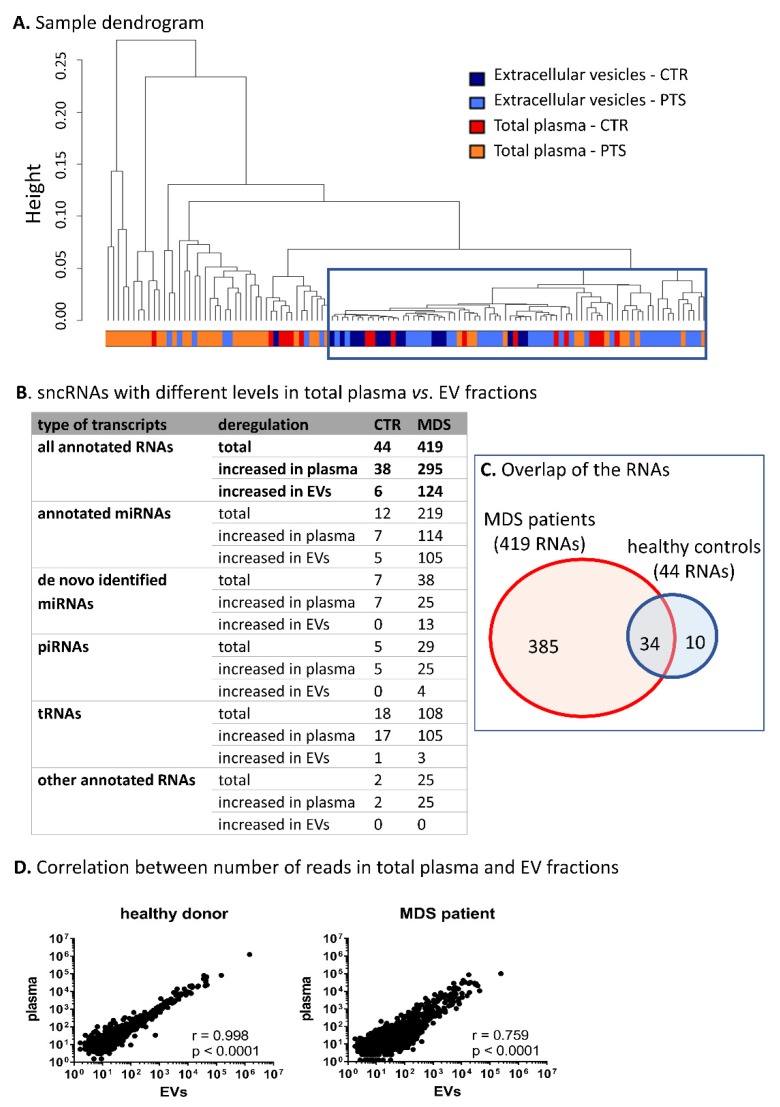
Characterization of circulating small noncoding RNA (sncRNA) profiles of total plasma vs. EVs. (**A**) Hierarchical cluster analysis of samples based on all RNA-seq data. The blue frame highlights the clustered extravesicular samples. CTR—healthy controls, PTS—MDS/AML patients. (**B**) SncRNAs with different levels between total plasma and paired EV samples. Only the RNAs with |logFC| >1 and *q* < 0.05 were considered. (**C**) The overlap of the RNAs between MDS patients and healthy controls. (**D**) Correlation between normalized number of reads in total plasma vs. paired EV samples in a typical healthy control or an MDS patient.

Hierarchical cluster analysis of all samples revealed that EV content was more homogeneous than the sncRNA content of total plasma, preferentially clustering the majority of EV samples into one cluster (Figure 2A). Furthermore, we performed differential expression analysis of paired samples of total plasma vs. EVs. Based on this comparison, we identified a striking difference between samples from MDS patients and those from healthy controls. The results showed a substantially higher number of differentially represented sncRNAs in MDS patients (419 sncRNAs) compared to healthy controls (44 sncRNAs) (plasma vs. EVs; |logFC| >1 and *q*-value <0.05, Figure 2B,D, the lists of deregulated sncRNAs are included in SI 9 and SI 10). The majority of the sncRNAs with different expression levels in controls were also found in MDS samples (Figure 2C); however, changes in the levels of 385 sncRNAs were uniquely identified only in MDS. It is of particular interest that levels of miRNAs were proportionally deregulated between the two materials (114 miRNAs were increased in total plasma and 105 miRNAs were increased in EVs), but the levels of piRNAs, tRNAs, and other RNA categories were almost exclusively increased in total plasma (25 piRNAs and 105 tRNAs were increased in total plasma and only four piRNAs and three tRNAs were increased in EVs) (Figure 2B).

### 2.5. SncRNAs Differentially Expressed in MDS

To characterize sncRNA profiles specific for MDS, we compared sncRNA levels between healthy controls, MDS patients, and AML-MRC patients. The results show relatively higher numbers of differentially represented sncRNAs (|logFC| >1 and *q* < 0.05) between MDS patients and healthy controls in total plasma (391 sncRNAs with 316 increased and 75 decreased sncRNAs) than in EVs (219 sncRNAs with 179 increased and 40 decreased sncRNAs). The lists of deregulated sncRNAs are included in SI 11 and SI 12, and SI 13A shows their distribution in various categories of transcripts. The overlap between the two groups comprised 130 sncRNAs (SI 14A). Importantly, we found that the levels of many hematopoiesis-related miRNAs were significantly increased in MDS patients compared to healthy controls, mostly both in plasma and EVs (e.g., miR-10a-5p, miR-29a-3p, miR-34a-5p, miR-99b-5p, miR-125a-5p, miR-146b-5p, and miR-150-3p/5p were increased both in total plasma and EVs, and let-7a-3p, miR-21-3p, miR-221-3p, miR-221-3p/5p, and miR-223-3p were increased only in total plasma; levels of several selected miRNAs are shown in SI 15). Regarding other types of sncRNAs, hsa_piR_019914/gb/DQ597347, hsa_piR_020450/gb/DQ598104, chr2.trna27-GlyCCC, chr18.trna4-LysCTT, SNORD119, and U33 were upregulated in MDS samples (SI 16).

On the other hand, there were almost no significant differences between sncRNA profiles of MDS and AML-MRC samples. There were only 9 and 14 differentially represented RNAs in plasma and EVs, respectively (more details are shown in SI 13B, SI 17, and SI 18).

### 2.6. SncRNAs Differentially Expressed in Different Stages of MDS

**Figure 3:**
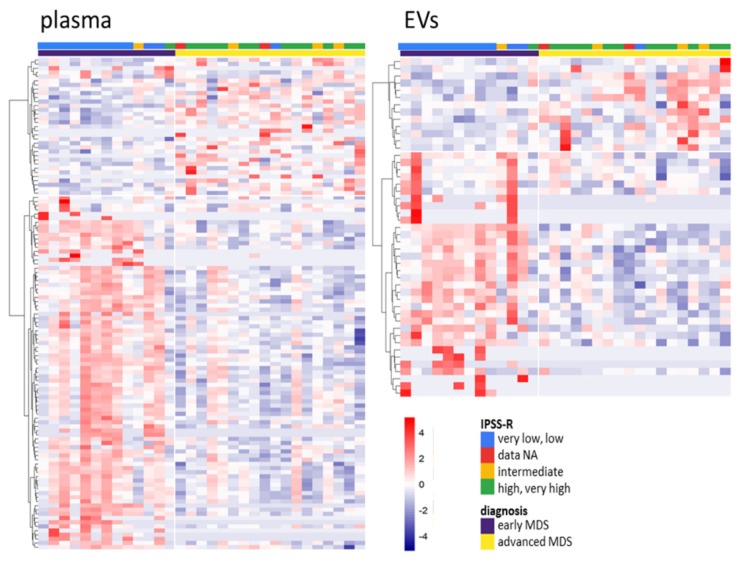
Heatmaps of differentially represented sncRNAs between early and advanced myelodysplastic syndromes (MDS) in total plasma (left) and EVs (right) (*q* < 0.05). The color gradient intensity scale shows the row z-score of counts per million (CPM; binary logarithm) of individual RNAs. Red indicates an increased level of an RNA, blue indicates a decreased level of an RNA.

To define sncRNAs with changed levels during MDS progression, we investigated the differences in sncRNA profiles between early (MDS-SLD, MDS-MLD, and MDS-5q-) and advanced stages of MDS (MDS-EB1 and MDS-EB2). The results showed 100 and 43 differentially represented sncRNAs (|logFC| >1 and *q* < 0.05) in plasma (81 increased and 19 decreased in early MDS) and EVs (34 increased and 9 decreased in early MDS), respectively. The SI 19 and SI 20 includes the complete list of deregulated sncRNAs, and SI 13C shows their distribution among various types of sncRNAs. The expression heatmaps in Figure 3 show the apparently distinct levels of these sncRNAs with regard to the stage of the disease when the patients are divided according to either WHO classification or IPSS-R score. 

The detailed examination of significantly deregulated sncRNAs (|logFC| > 1 and *q* < 0.05) between early and advanced stages of MDS revealed that only 14 sncRNAs were deregulated in both types of material, total plasma and EVs (SI 14B). Regarding hematopoiesis-related miRNAs (e.g., miR-103a-3p, miR-103b, miR-107, miR-221-3p, miR-221-5p, and miR-130b-5p), their levels were significantly decreased in total plasma of advanced MDS compared to early MDS patients (SI 21). Interestingly, multiple miRNAs (e.g., miR-127-3p, miR-154-5p, miR-323b-3p, miR-382-3p, miR-409-5p, and miR-485-3p) clustered in chromosomal region 14q32 were found among the significantly upregulated miRNAs in total plasma and/or EVs of early MDS (SI 22). Regarding other sncRNA species, hsa_piR_000805/gb/DQ571003, hsa_piR_019420/gb/DQ596670, chr6.trna152-ValCAC, and chr7.trna5-CysGCA were significantly deregulated between early and advanced MDS (SI 23).

### 2.7. Validation of NGS Results

We evaluated the levels of miR-16-5p, miR-34a-5p, miR-125a-5p, miR-125b-5p, miR-127-3p, miR-221-3p, and hsa_piR_001170/DQ571526 in an independent cohort of samples comprising 36 MDS patients, 7 AML-MRC patients, and 12 controls, SI 4. These sncRNAs were selected based on their previously described relevance for MDS and/or significantly different levels in the small RNA-seq experiments. After absolute quantification of sncRNA levels by ddPCR, we compared these results with those of the small RNA-seq analysis by performing Pearson correlation of the mean expression values measured in the individual sample groups (healthy controls, early MDS, advanced MDS, and AML-MRC). The correlation analysis proved concordance of both methods (r = 0.606, *p* = 0.0001, SI 24).

### 2.8. Pathway Analysis Based on miRNA Profiles Specific for MDS

To identify biological functions potentially influenced by deregulated miRNAs circulating in MDS plasma, we performed miRNA target prediction coupled with pathway enrichment analysis. The analyses were performed for miRNAs differentially represented (|logFC| >1, *q* < 0.05) between MDS vs. control samples and between early vs. advanced MDS samples either in plasma or EVs. As shown in Table 1 in more detail, we found that the deregulated miRNAs were associated with multiple pathways related to cancer (namely, the Ras, TGF-beta, ErbB, and Rap1 pathways), pluripotency of stem cells, extracellular matrix (ECM), and focal adhesion.

### 2.9. Relationship of Somatic Mutations and Levels of Circulating sncRNAs in MDS

We explored the possible relationship between the presence of somatic mutations and the levels of circulating sncRNAs in MDS. Using differential expression analyses, we searched for sncRNAs with differential expression between MDS patients with vs. without a mutation in the *SF3B1* or *DNMT3A* genes. The analysis identified only a few deregulated sncRNAs with *q* < 0.05 (*SF3B1*: no sncRNAs, *DNMT3A*: miR-7515 in total plasma, miR-6857-3p, miR-9-3p, and hsa_piR_020485/gb/DQ598159 in EVs, SI 25A).

To gain better insight into the potential effects of *SF3B1* and *DNMT3A* mutations on the levels of circulating sncRNAs, we refined the selection criteria and included all sncRNAs at raw *p* < 0.01 (non-adjusted for multiple testing) with the awareness of potential high numbers of false positive results. Regarding the *SF3B1* mutation, we identified 22 and 15 sncRNAs deregulated in total plasma and EVs, respectively. The *DNMT3A* mutation was associated with the deregulation of 34 sncRNAs in total plasma and 32 sncRNAs in EVs (SI 26). Finally, we intersected deregulated sncRNAs in the two materials and found that total plasma and EVs displayed different sncRNA profiles. Only miR-100-5p and miR-450b-5p of *SF3B1*-mutated samples were deregulated in both materials, plasma and EVs (SI 25B).

### 2.10. A-to-I Editing of Circulating miRNAs

Within the small RNA-seq data, we also characterized A-to-I editing profiles of circulating miRNAs in MDS. RNA editing is a phenomenon when a single-nucleotide change occurs (mainly adenosine to inosine modification, A-to-I) in a miRNA sequence and in turn, it can impact the biogenesis and specificity of mature miRNAs. In the read sequences, we identified 38 A-to-I editing events in 32 miRNAs that were detected in more than 2% in at least one sample; however, the overall editing level was quite low. Interestingly, samples of early MDS patients in both total plasma and EVs showed higher numbers of editing events compared to those of healthy controls and advanced MDS/AML-MRC patients. On average, 10.0 editing events per sample were found in early MDS patients compared to 3.7 and 5.4 events per sample in healthy controls and advanced MDS/AML-MRC, respectively (SI 27A).

With respect to the potential biological relevance of such low numbers of edited sequences from the total number of reads, we further focused the analysis only on the main editing events (i.e., editing found in ≥10 samples with level ≥5%). Only 11 miRNA editing events remained after the exclusion of low-editing data (miR-99a-5p, miR-206, miR-337-5p, miR-369-5p, miR-376c-3p, miR-379-5p, miR-381-3p, miR-411-5p, miR-485-3p, miR-497-5p, and miR-664a-5p, SI 27B and 27C). Among them, the editing levels of two miRNAs, miR-376c-3p and miR-411-5p, were significantly increased in both total plasma and EVs in MDS patients compared to controls, irrespective of the stage of the disease (SI 27D). However, given the limited number of editing events detected and their low rates compared to wild type sequences, we identified no significant editing changes in individual miRNAs with respect to the stage of the disease or in comparison to AML-MRC, thus not assigning an important role of A-to-I editing in circulating miRNAs in myelodysplasia.

### 2.11. Circulating sncRNAs as Prognostic Biomarkers of MDS Survival

The RNA-seq data were subjected to survival analysis, and two sets of sncRNAs (separately for the two materials, total plasma and EVs) whose levels significantly correlated with OS were identified. Of the 3130 sncRNAs that were uploaded into the analysis tool, 173 and 122 sncRNAs were significantly (univariate analysis *p* < 0.05) associated with OS in total plasma and EVs, respectively (SI 28 and SI 29). From these, only the sncRNAs with the highest level of association (permutation *p* < 0.001) were chosen for further analyses, namely, 3 sncRNAs in total plasma (miR-1260b, miR-3191-3p, and miR-328-3p) and 4 sncRNAs in EVs (miR-1237-3p, U33, hsa_piR_019420/gb/DQ596670, and miR-548av-5p) (the results are summarized in Table 2 and Kaplan–Meier plots are shown in SI 30).

To test a combination strategy of multiple survival-associated sncRNAs for better patient stratification, we defined a risk prediction score that combined the effects of the selected sncRNAs above. The coefficients of survival risk formula for these individual sncRNAs contributing to final risk assessment and *p*-values of cross-validation tests are included in Table 2. The final survival risk score of a total plasma sample was calculated based on the following formula:

Plasma risk score = −0.631 × log_2_ (level of miR-1260b) − 0.24 × log_2_ (level of miR-328-3p) + 6.861.

Similarly, the survival risk score of an EV sample was calculated as follows:

EV risk score = 0.615 × log_2_ (level of miR-1237-3p) + 0.917 × log_2_ (level of U33) − 0.106 × log_2_ (level of hsa_piR_019420) − 1.01 × log_2_ (level of miR-548av-5p) − 4.948.

A higher score (>0) indicated an increased risk of mortality, whereas a lower score (≤0) denoted a better outcome.

**Figure 4:**
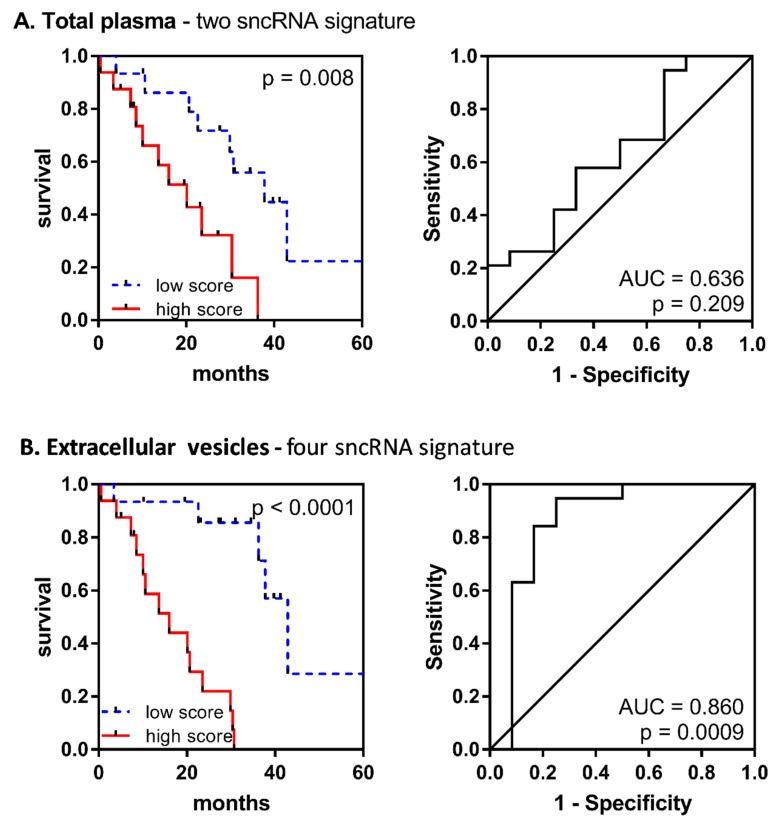
Performance of the combined prognostic model for the overall survival of MDS patients. Kaplan–Meier curves and receiver operation characteristic (ROC) curves are shown for (**A**) a two-sncRNA signature (miR-1260b and miR-328-3p) in total plasma and (**B**) a four-sncRNA signature (miR-1237-3p, U33, hsa_piR_019420, and miR-548av-5p) in EVs.

To evaluate the performance of combined prognostic signatures, Kaplan–Meier curves and receiver operation characteristic (ROC) curves were plotted for the samples divided into high- and low-risk groups according to the computed score formulas. The results showed that combining sncRNA level scores increased the predictive power of the survival risk model more significantly in EV material (univariate *p* < 0001, ROC: AUC = 0.860, *p* = 0.0009) than in total plasma (univariate *p* = 0.008, ROC: AUC = 0.636, *p* = 0.206) (Figure 4).

Furthermore, we tested the relationship of individual survival-associated sncRNAs and their combination scores with clinical parameters. A series of Pearson correlation tests showed a significant association between the levels of the majority of these sncRNAs and bone marrow blast count and platelet count (SI 31). However, Cox multivariate analysis revealed that the EV combined score (HR = 5.866, 95% CI 2.262 to 15.210, *p* < 0.001) was the variable most significantly associated with OS, even when compared with the IPSS-R score (HR = 1.410, 95% CI 0.840 to 2.366, *p* = 0.193) (Table 3).

### 2.12. Circulating sncRNAs Predictive of the AZA Response

To search for sncRNAs applicable as predictive biomarkers of the AZA response, we analyzed RNA-seq data from AZA-treated MDS/AML-MRC patients. Using differential expression analysis, we found only a few sncRNAs significantly (|logFC| >1, *q* < 0.05) associated with patient response to AZA. In total plasma, the levels of miR-4774-3p and miR-762 were increased, and the levels of miR-125b-5p, miR-4324, miR-3156-5p, and miR-3692-3p were decreased in relation to a later response to AZA treatment. In EVs, different sncRNAs were associated with AZA response; levels of miR-6857-3p, miR-1299, miR-183-5p and miR-513b-3p were increased, and miR-6832-3p levels were decreased (SI 32).

**Figure 5:**
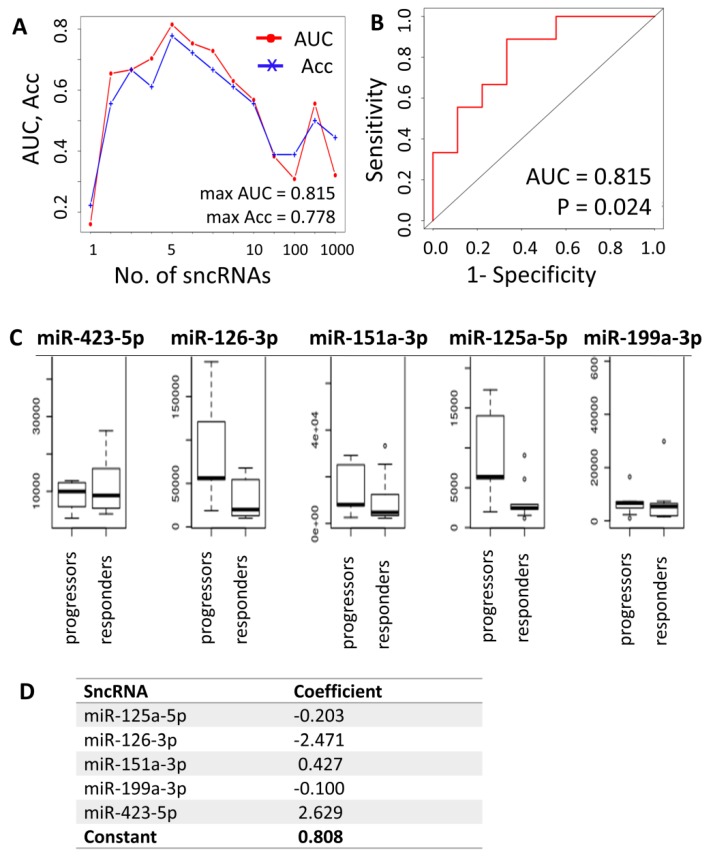
Combined prediction model for response to agent azacytidine (AZA) treatment in MDS/AML-MRC patients. (**A**) Results of the support vector model support vector machine-recursive feature elimination (SVM-RFE) regression model determining the optimal number of sncRNAs whose combined expression could be predictive of the likelihood of response. (**B**) ROC curve for the five sncRNA predictors. (**C**) Plasma levels of the best/most common predictors (miR-423-5p, miR-126-3p, miR-151a-3p, miR-125a-5p, and miR-199a-3p). (**D**) Output of the sag solver analysis for calculation of predictive formula for AZA treatment response. Predictive formula: score = 2.629 × ln(level of miR-423-5p) − 2.471 × ln(level of miR-126-3p) + 0.427 × ln(level of miR-151a-3p) − 0.203 × ln(level of miR-125a-5p) − 0.1 × ln(level of miR-199a-3p) + 0.808. A score >0 predicts future response to AZA, whereas a score ≤0 predicts disease progression despite AZA treatment. AUC—area under the ROC curve, Acc—accuracy.

Because the results of differential expression analysis were limited to only several miRNAs of low predictive value, we performed additional machine learning analysis to define a combined sncRNA signature that would predict the AZA response with higher accuracy compared to individual sncRNAs. First, the data were subjected to the support vector machine support vector machine-recursive feature elimination (SVM-RFE) regression model, which showed that the best classification of responders vs. progressors could be achieved using cumulative expression data of five sncRNAs measured in total plasma (AUC = 0.815, Acc = 0.778), with 6 to 10 sncRNAs combined being of less predictive value (Figure 5A,B). The five most common sncRNAs with the best cumulative predictive value determined by SVM were miR-423-5p, miR-126-3p, miR-151a-3p, miR-125a-5p, and miR-199a-3p (Figure 5C). However, these results were achieved only for the total plasma and for the clearly defined groups of patients (responders vs. progressors). When testing the data from EV samples or trials involving patients with stable disease after AZA treatment, no significant differences were detected.

The sncRNAs preselected by differential expression analysis (miR-125b-5p, miR-4324, and miR-4774-3p) and the sncRNAs with the best cumulative predictive value in the SVM model (miR-423-5p, miR-126-3p, miR-151a-3p, miR-125a-5p, miR-199a-3p, miR-142-5p, Ro-associated RNA, miR-185-5p, miR-30d-5p, miR-92a-3p, let-7a-5p, let-7f-5p, and miR-26b-5p) for total plasma samples of responders vs. progressors were further statistically tested together by logistic regression analysis using maximum likelihood estimation, and their number was reduced to five (miR-423-5p, miR-126-3p, miR-151a-3p, miR-125a-5p, and miR-199a-3p), which was in agreement with the SVM method. With these five miRNAs, the following predictive formula was calculated (Figure 5D):

Prediction score = 2.629 × ln(level of miR-423-5p) − 2.471 × ln(level of miR-126-3p) + 0.427 × ln(level of miR-151a-3p) − 0.203 × ln(level of miR-125a-5p) − 0.1 × ln(level of miR-199a-3p) + 0.808.

A score >0 predicted future response to AZA, whereas a score ≤0 predicted progression of the disease despite AZA treatment. The quality of the prediction was 88.9% (16 out of 18 patients).

## 3. Discussion

At the beginning of this millennium, the first extracellular sncRNAs circulating in blood were discovered [21]. Since then, a wide variety of RNA species have been found in blood plasma [22,23] and its extracellular vesicles [24] by application of high-throughput sequencing. As diagnostic and therapeutic procedures shift from biopsies towards less invasive techniques, sncRNAs circulating in the blood become of particular interest as potential blood-based biomarkers. Therefore, further characterization of circulating sncRNAs is needed to provide reference profiles for the development of biomarkers of human diseases. In the present study, we investigated sncRNA profiles in paired samples of total plasma and its EV fraction obtained from MDS and AML-MRC patients and healthy controls to evaluate the potential of circulating sncRNAs to become novel noninvasive biomarkers able to improve current stratification systems in MDS diagnosis.

It has been recognized that tumor cells secrete significantly more exosomes into the tumor microenvironment than normal cells, leading to an increase in exosome levels in the circulatory system [25]. Szczepanski et al. showed that the serum of AML patients contained higher levels of microvesicles compared to that of healthy controls [26]. In MDS plasma, we did not identify higher numbers of EVs but rather noticed differences in their sizes, specifically a particular increase in EVs of larger diameters and a higher content of their RNA cargo (SI 5). This is in agreement with a previous study showing that the RNA content was doubled in MDS compared to controls [19]. Thus, functional studies investigating reasons of this increase and its relation to MDS pathogenesis are needed.

The annotation process of circulating RNAs revealed that more than a third of reads were not mappable to the human genome. The majority of these reads mapped to nonhuman rRNA sequences, and the most frequently represented rRNAs originated from the phylum *Proteobacteria* (SI 8). Consistent with these findings, it was previously shown that a significant fraction of the circulating RNAs appear to originate from exogenous species and are of a rRNA origin [27]. The blood microbiome is predominated by *Proteobacteria*, *Actinobacteria*, *Firmicutes,* and *Bacteroidetes* [28]. Importantly, it has been demonstrated that some of these RNAs may influence cellular activities under in vitro conditions. For example, Buck et al. demonstrated that exosomes secreted by gastrointestinal nematode parasites can transfer sncRNAs to mammalian cells to modulate host innate immunity [29]. This raises the possibility that plasma RNAs of exogenous origin may serve as signaling molecules reflecting the state of human health and that they may also affect features of hematopoietic cells, contributing to the pathogenesis of blood diseases. Within the scope of this study, we further investigated only sncRNAs of human origin; however, circulating microbial RNAs may also bring large, so far uncovered potential as new biomarkers for the diagnosis and prognosis of myeloid diseases.

Hierarchical clustering of all samples based on their RNA profiles showed that EV cargo is more homogeneous than the sncRNA content of total plasma (Figure 2). The larger heterogeneity of sncRNAs in plasma can be expected because blood plasma carries a mixture of various RNA transporters (i.e., proteins, lipoprotein particles, different microvesicles, and apoptotic bodies), whereas the EV fraction contains mostly purified exosomes. Moreover, it has been demonstrated that specific small RNAs are packaged in EVs in a selective way [30], which may contribute to the higher level of homogeneity of the EV fraction. Thus, the data suggest that the contents of the two materials do not completely reflect one another but rather represent independent sources of RNA, providing different insights into the biological processes occurring during disease pathogenesis.

Because of the variations in RNA profiles between total plasma and its EV fraction, we further tested both of these materials in parallel to assess which of them could provide better performance as a source of circulating RNA biomarkers applicable for MDS stratification. Because the majority of published studies have focused only on one type of material, not directly comparing total plasma with EVs, there is still no consensus on the appropriate source of these biomarkers.

Subsequent classification of read sequences into various RNA categories revealed additional differences between the two types of tested material. Samples of total plasma had a substantially higher proportion of miRNA reads (60% of miRNAs in total plasma vs. 46% in EVs) and a lower proportion of uncharacterized reads (33% of these reads in total plasma vs. 48% in EVs) (Figure 1). It is still debated whether EVs contain biologically meaningful amounts of miRNAs able to provide a source of miRNA biomarkers. Chevillet et al. suggest that most individual exosomes do not carry any biologically significant numbers of miRNAs and are, therefore, unlikely to be of physiologic relevance in miRNA-based cell-to-cell communication [31]. A significant underrepresentation of miRNAs over other RNA species in exosomes has been confirmed by other studies [30,32,33]. On the other hand, there are studies demonstrating that exosomes provide a sufficient source of miRNA for disease biomarker detection [34,35].

Within differential expression analyses, we found substantial variability between the two types of material on the level of individual sncRNAs. One of the most interesting findings of this study was the fact that the RNA profiles of total plasma and paired EVs substantially differed between MDS patients, whereas they remained closely similar in healthy individuals (Figure 2). Such a high number of sncRNAs with changed levels in MDS (almost 10-fold increase) suggests that mechanisms of sncRNA export into the blood circulation may be specifically affected in MDS, changing sncRNA profiles as a whole. Other studies also found no significant difference between plasma and exosomal miRNAs from healthy people [36]. On the other hand, differential regulation of miRNA levels between plasma and exosomes in different disease animal models has been described as well [37]. Therefore, it seems that export of sncRNAs into blood circulation may be specifically affected in various disease states. As a result, these changes might affect cell-to-cell communication of blood cells, potentially contributing to disease pathogenesis.

Moreover, we noticed that differentially represented miRNAs were proportionally deregulated between the two materials in MDS patients, whereas other sncRNA species, such as piRNAs and tRNAs, were almost exclusively increased in MDS plasma compared to paired EVs. These disproportions might indicate that miRNAs probably use at least partly different mechanisms of export than mechanisms exploited for other non-miRNA species of sncRNAs and that these mechanisms may be affected in MDS in different ways. Other studies have also demonstrated that different sncRNA species are preferentially loaded into different types of carriers [33,38] and that packaging of specific RNA molecules into carriers is selective within individual RNA species [37,39].

To characterize the MDS-specific profile of circulating sncRNAs, we compared their extracellular levels in MDS samples with those in AML-MRC and healthy controls. We found hundreds of differentially represented sncRNAs between MDS and control samples in both plasma and EVs, indicating physiological changes related to the disease. However, we found almost no significant differences in sncRNA profiles between MDS and AML-MRC samples. It has been previously noted that there are significant biological and clinical differences between AML and MDS, and based on these observations, it has been warranted that MDS should not be considered only as an early phase of AML or as preleukemia [40]. However, based on the link between MDS and AML-MRC in terms of potential pathways and genetic biomarkers [41,42,43] and similar molecular characteristics described here and in several preceding papers, it is evident that MDS and AML-MRC share important features on the biological level, enabling the application of similar therapeutic approaches that specifically address both of these pathological entities.

To obtain a better insight into changes of molecular profiles specific for MDS in comparison with healthy controls, we focused on the levels of individual miRNAs and observed that the majority of them were increased in both plasma and EVs of MDS patients. Among these, we found many miRNAs whose intracellular deregulation has already been associated with various hematopoietic disorders, including MDS, such as miR-34a, miR-125a, miR-99b, miR-10a, miR-221, miR-222, miR-223, miR-29a, and miR-150 [44,45]. Although the physiological roles of circulating miRNAs and the impact of their deregulation are still unclear, there is convincing evidence of their intracellular regulatory functions in hematopoiesis and its pathogenesis.

For example, miR-34a is a tumor suppressor directly regulated by p53, and its transactivation broadly influences gene expression and promotes apoptosis [46]. Overexpression of miR-34a in early MDS has already been described in preceding studies and is attributed to the increased apoptosis occurring in these patients [42,47,48]. Another study demonstrated that the overexpression of miR-34a in MDS granulocytes reduces level of c-Fos resulting in excessive production of TNF-α contributing to the development of ineffective hematopoiesis [49]. Furthermore, miR-125a is highly expressed in hematopoietic stem cells (HSCs) and controls their numbers [50]. miR-125a knockout mice were shown to develop myeloproliferative disorders [51]. Upregulation of miR-125a and miR-99b in macrophages leads to their polarization and secretion of inflammatory cytokines to kill tumor cells [52]. miR-10a regulates myeloid differentiation, and its level is increased in AML [53] and atypical myeloproliferative neoplasms [54]. miR-221 and miR-222 play a suppressing role in erythroid differentiation [55] and are consistently overexpressed in AML [56]. miR-223 is suppressed in AML patients but is able to inhibit cell proliferation and enhance cell apoptosis in AML cell lines [57]. Another miRNA involved in AML development is miR-29a, a key regulator of normal myeloid differentiation with tumor-suppressive function [58]. miR-29a is highly expressed in normal HSCs, inducing their self-renewal [59]. miR-150 regulates B and T-cell differentiation and maturation [60,61]. Increased miR-150 expression contributes to myelodysplastic hematopoiesis in MDS-del(5q) via its negative regulation of the transcription factor MYB [62]. In conclusion, it may be possible that a big increase in these miRNAs in blood circulation could have a significant impact on multiple recipient cells, affecting their physiological features and contributing to the development of myelodysplasia.

To obtain an additional look at biological functions that could be affected by deregulated miRNAs in MDS, we performed a miRNA target prediction followed by pathway enrichment analyses. Although different miRNAs were deregulated in total plasma vs. EVs, considerable similarities in the affected pathways were observed, pointing to the shared targets of different miRNAs. Several signaling pathways associated with cancer and pluripotency of stem cells were identified as potentially targeted by the deregulated miRNAs (i.e., the Ras, TGF-β, and ErbB pathways). Furthermore, the deregulated miRNAs were also associated with pathways related to the extracellular environment and cell interactions (ECM-receptor interactions, focal adhesion, and proteoglycans in cancer). ECM is a complex network of extracellular macromolecules that provides support for surrounding cells. Specific interactions between cells and the ECM are mediated by transmembrane and cell-surface-associated components, leading to the control of different cellular activities, such as adhesion, migration, differentiation, proliferation, and apoptosis. Proteoglycans have been shown to be key macromolecules that contribute to the biology of various types of cancers through the abovementioned processes [63]. Exosomes can also be considered integral components of the ECM since they modulate the assembly of the molecular network and signaling through the ECM [64]. The pathway analysis thus suggested that the miRNAs released into blood circulation in MDS patients may further potentiate dysregulation of biological processes in the extracellular environment. The miRNAs might even act as a kind of regulatory loop affecting the functionality of EVs themselves and/or facilitating changes in the hematopoietic niche microenvironment.

Somatic mutations in multiple genes have recently been described in MDS and are rapidly becoming the most frequently discussed aberrations associated with MDS [65]. Therefore, we investigated the possible link between mutations in *SF3B1* or *DNMT3A* genes and the export of sncRNAs into blood circulation. However, only low numbers of circulating sncRNAs were significantly related to the mutational status of patients. This raises the question of whether the effects of a somatic mutation in a single gene on sncRNA export are either negligible or whether the analysis was affected by a bias. Regarding possible bias, a low number of patients with detected mutations was analyzed. Moreover, the data were undoubtedly influenced by substantial heterogeneity on multiple levels (i.e., additional presence of various cytogenetic aberrations that are more penetrant, differences in variant allele frequencies, cooccurrence of several mutations in a single patient, or a wide spectrum of variants in one gene that may differentially influence protein activity). In conclusion, although significant bias could affect the validity of the results, only slight trends in differential patterns of circulating sncRNAs with relation to the mutational status have been observed, suggesting that there is no fundamental association of somatic mutations in *SF3B1* or *DNMT3A* genes and sncRNA export from MDS cells.

In addition to expression level analysis, variation in sequences, such as RNA editing, can also be detected by the RNA-seq method. Very recently, Nigita et al. found that miRNA editing events also occur in blood circulation, and the analysis of exosomal edited cargo was able to distinguish between normal and tumor sample subtypes [66]. In MDS, a pilot study on RNA editing was published by Crews et al., who showed that A-to-I RNA editing rates were increased in AML-MRC compared to MDS progenitors and that the differential expression of certain sites was as high as 70% [67]. However, we identified only a limited number of edited miRNAs with low rates of A-to-I changes compared to wild type miRNAs (SI 18). Unlike the abovementioned publication, we detected an increase in editing events in circulating miRNAs of early MDS, whereas the editing rates in advanced MDS and AML-MRC samples remained similar to those of healthy controls. These are only preliminary data on the possible significance of RNA editing in the development of MDS that must be considered within a larger context in subsequent studies. Here, we conclude that although we identified some miRNAs with altered editing in MDS plasma, the biological relevance of the RNA editing process is questionable given the low rates found.

Because peripheral blood is available almost noninvasively compared to solid tissue biopsies, blood-based biomarkers become of particular interest as a promising noninvasive strategy for the classification of different cancers. Thus, we aimed to define novel circulating sncRNA-based biomarkers that are able to predict the outcome of MDS patients. To assess changes in circulating sncRNA profiles associated with MDS progression, we compared sncRNA profiles in early and advanced MDS. In both materials, plasma and EVs, 80% of the total amount of deregulated sncRNAs in MDS was specifically increased in early MDS compared to advanced MDS. This difference might be linked to excessive apoptosis in early MDS and subsequent switching to a pro-proliferative phenotype accompanied by inhibition of the apoptotic process in advanced MDS [68]. Recent studies have shown that specific extracellular vesicles, termed “apoptotic exosome-like vesicles” (AEVs), are released from apoptotic cells. These AEVs appear to be more than just debris and should be considered a key mechanism for apoptotic cells to communicate with surrounding cells. Moreover, they have important immune regulatory roles that differ from the functions of classical EVs [69,70] and might be relevant for the pathogenesis of various inflammatory diseases, including autoimmune diseases, cancers, and even MDS.

When exploring evidence for an association with apoptosis, we found that multiple miRNAs from a large cluster located within the 14q32 locus were significantly upregulated in early MDS. These miRNAs are frequently altered in various cancers, and their deregulation has been linked to abnormal induction of apoptosis and suppression of proliferation. They are also involved in HSC differentiation [71]. The upregulation of miRNAs within the 14q32 locus has already been reported in MDS by several studies [47,48,72]. In our recent study, we demonstrated increased expression of 14q32 miRNAs in CD34+ cells in advanced stages of MDS and in AML-MRC and associated this elevation with poor outcome. Moreover, the increased intracellular levels of 14q32 miRNAs were reduced after AZA treatment [73]. Based on the present data, it seems that the levels of 14q32 miRNAs have opposite trends in HSCs and in plasma in different stages of the disease, i.e., these miRNAs are released into the extracellular environment in early, pro-apoptotic stages of MDS but are retained intrinsically along with disease progression.

Although we associated some miRNAs with MDS progression, we intended to identify the biomarkers with the highest predictive values among the various RNA molecules circulating in MDS patient blood. Therefore, we performed an additional series of bioinformatic analyses of RNA-seq data with regard to the survival of MDS patients and their response to AZA treatment. We identified several sncRNAs whose extracellular levels were strongly predictive of patient outcome. However, we almost completely failed to predict the response to AZA treatment on the level of individual sncRNAs. The predictive value of circulating sncRNAs (for prediction of both patient survival and response to the treatment) was substantially increased by the generation of combined panels of specific sncRNAs. More importantly, multivariate analysis proved that the combined sncRNA panel for EV samples was associated with patient outcome more significantly than the clinical variables typically used for routine MDS diagnostics, which clearly points to the considerable potential of these methods for better stratification of MDS patients. The five most common sncRNAs with the best cumulative predictive value were miR-126-3p, miR-125a-5p, miR-199a-3p, miR-151a-3p, and miR-423-5p. It was found that miR-126 inhibits cell apoptosis and increases viability of AML cells in vitro [74] and thus, may function as an oncogene in leukemogenesis. Higher expression level of miR-126-3p was correlated with poorer prognosis in AML patients [75]. miR-125a knockout mice were shown to develop myeloproliferative disorders [51]. In an in vitro study, miR-125a-5p induced granulocytic differentiation in different human AML cell lines as well as in normal human primary hematopoietic progenitor/stem cells [76]. Another study showed that enforced expression of miR-199a-3p enhanced proliferation of myeloid progenitor cells. In addition, miR-199a-3p caused AML in a pre-leukemic mouse model, supporting its role as an onco-microRNA. The target genes of miR-199a-3p included *PRDX6*, *RUNX1*, and *SUZ12* [77]. miR-151-3p and miR-423-5p were observed to be involved in regulation of different solid tissue cancers and other diseases, however, their role in hematology is yet unknown. Although these results suggest the considerable potential of circulating sncRNAs as novel noninvasive biomarkers in MDS prognostication, we are aware that the data have to be validated on larger independent cohorts of patients before their inclusion into routine clinical practice.

In addition to miRNAs, we identified a large set of other sncRNA species deregulated in our MDS cohort; however, little is known about non-miRNA sncRNA species in blood circulation. So far, the most highlighted are piRNAs and tRNAs, as they are quite abundant within small RNA-seq studies. piRNAs were originally described as key functional regulators for germline maintenance [78] and transposon silencing [79], but recent evidence has revealed that piRNA expression differs substantially across somatic tissues and that their aberrant expression is a unique feature in many diseases, including multiple human cancers [80]. Recently, tRNA-derived small RNAs (tsRNAs), a new class of sncRNAs, have been found. In addition to their well-known function in protein translation, they function in regulating responses to different types of stress [81], participate in gene silencing [82], modulate gene expression [83] and have a specific impact on cancer development [84,85,86]. It is unclear if the levels of tsRNAs detected in RNA-seq experiments represent the full-length tRNA levels, if they are just physiologic byproducts that reflect the level of tRNA processing or if they are biologically active entities themselves. To date, only two studies addressing non-miRNA sncRNA species in MDS have demonstrated their utility as prognostic biomarkers in MDS [87,88]. Here, we showed that miRNAs reached higher predictive value for both prediction of patient outcome and therapy response compared to other types of sncRNAs. Therefore, utilizing miRNAs as possible biomarkers is still the most meaningful approach under the present circumstances. However, further exploration of other sncRNA species is essential to represent real conditions and to bring new insight into sncRNA functions in health and disease.

One of the major aims of this study was to define what type of material, total plasma or its EV fraction, would be more suitable for the measurement of circulating biomarkers of MDS. In the search for sncRNA-based biomarkers of MDS, we found that EVs were a better source of sncRNA biomarkers for patient survival, whereas sncRNAs circulating in total plasma were predictive of AZA treatment. In summary, our results suggest that the two materials represent distinctive snapshots of the disease taken from two different points of view and may not be replaced by one another. This is in agreement with the results of another study comparing miRNA content in total plasma and plasma-derived exosomes in kidney disease [37]. The authors showed that miRNAs in plasma and in exosomes are differentially regulated and thus the measurement of exosomal miRNAs cannot be replaced by the measurement of miRNAs in plasma, or vice versa [37].

Based on the observations we made within this study, we can conclude that the possible utilization of circulating sncRNAs as biomarkers in MDS seems to be meaningful. We showed that deregulation of the levels of various RNA molecules present in blood circulation reflects the actual stage of the disease. However, improved knowledge of biological changes with respect to sncRNA export, due to its altered dynamics in health and disease conditions, is still needed to have relevant implications for disease detection and treatment. Moreover, it remains to be elucidated whether changes in circulating RNA levels are a consequence of cellular disorders related to a disease without any specific function, when its impact on recipient cells would be just an accident, or if there is a purpose of affecting specific recipient cells in a particular way. Recent investigation shows that high levels of RNAs circulating in human blood likely have a substantial impact on processes in recipient cells. The effects of sncRNAs taken up by recipient cells could lead either towards improvement or further induction of the disease. For example, Hornick et al. observed that the unique miRNA profile of AML exosomes has the potential to increase leukemic fitness by dysregulating other cell types [89].

To conclude, our findings suggest that the RNA export pattern changes along with myelodysplasia, causing differences in the levels of many hematopoiesis-related miRNAs as well as other classes of sncRNAs. These data not only provide a rationale for the potential clinically useful application of circulating sncRNAs in the prognosis of MDS but also raise new intriguing questions about the pathobiology of export mechanisms and possible consequences of their defects in hematopoietic disorders.

## 4. Methods

The main study cohort included PB plasma samples from 31 MDS and 11 AML-MRC patients examined by small RNA-seq (SI 1). An independent validation cohort included 36 MDS and 7 AML-MRC patients (SI 4) and was analyzed by droplet digital PCR (ddPCR). As controls, PB plasma samples from 29 age-matched healthy donors (17 individuals analyzed by small RNA-seq and 12 individuals by ddPCR) with no adverse medical history were used. Written informed consent was obtained from all tested subjects in accordance with the approval from the Institutional Review Board.

Plasma was separated from PB by centrifugation. EV fractions were extracted from plasma using the ExoQuick Plasma Prep and Exosome precipitation kit (System Biosciences, Palo Alto, CA, USA). To achieve efficient recovery of EVs, the plasma samples were pretreated with thrombin (5 U/mL) to defibrinate the samples and then EVs were extracted from 250 µL of defibrinated plasma according to the manufacturer’s protocol. To confirm the presence and size of EVs, transmission electron microscopy (TEM), nanoparticle tracking analysis (NTA), and Western blotting were performed.

Total RNA was extracted using the miRNeasy Serum/Plasma Kit (QIAGEN, Hilden, Germany). Small RNA-seq libraries were constructed using a QIAseq miRNA Library Kit (QIAGEN) and sequenced on a HiSeq 2500 sequencer (Illumina, San Diego, CA, USA). Sequences were subsequently processed using the QIAseq miRNA Primary Quantification pipeline (QIAGEN). De novo miRNAs were predicted using the miRdeep2 tool. The remaining reads unmapped to the human genome were analyzed by the metagenome analyzer MEGAN [90]. Analysis of A-to-I editing was performed using the miRge2.0 tool [91]. Data normalization and subsequent statistical analyses were performed using the edgeR package [92] in the R statistical environment. Binary logarithms of fold changes (logFC) and *q*-values (false discovery rate (FDR) adjusted *p*-values) were generated as an output of edgeR package for differential expression analysis of the data.

Mutational screening was performed using the TruSight Myeloid Sequencing Panel Kit (Illumina). The libraries were sequenced on a MiSeq instrument (Illumina), and the data were analyzed using NextGENe software (SoftGenetics, State College, PA, USA).

The quantity of individual miRNAs (miR-16-5p, miR-34a-5p, miR-125a-5p, miR-125b-5p, miR-127-3p, miR-221-3p, and hsa_piR_001170/gb/DQ571526) was verified via ddPCR using a QX200 ddPCR system (Bio-Rad, Hercules, CA, USA) with a TaqMan MicroRNA Reverse Transcription Kit and TaqMan miRNA assays (ThermoFisher Scientific, Waltham, MA, USA) in an independent cohort of 55 samples (SI 4). 

The OS-associated sncRNAs were identified by performing univariate Cox regression along with a permutation test using BRB-ArrayTools [93]. Multivariate Cox regression analysis was performed to identify independent variables associated with patient survival. A support vector machine (SVM) regression model was used to define sncRNA classifiers that discriminated AZA responders and nonresponders. Other statistical analyses were performed using GraphPad Prism v7 software (GraphPad Software, La Jolla, CA, USA).

Pathway analysis was performed based on significant differences in miRNA levels using DIANA-miRPath v3.0 [94].

A detailed version of the Methods is included in the manuscript as a Supplementary file (SI 33, Supplementary Methods).

## Figures and Tables

**Table 1:** The most significantly enriched pathways in the four different sets of deregulated miRNAs. The top ten pathways with the highest *p*-values are listed for each dataset.

KEGG Pathway	*p*-Value
plasma: MDS vs. CTR	
Mucin type O-Glycan biosynthesis	9.77 × 10^−15^
Proteoglycans in cancer	6.05 × 10^−9^
ErbB signaling pathway	2.80 × 10^−8^
Ras signaling pathway	2.02 × 10^−7^
Axon guidance	2.02 × 10^−5^
Pathways in cancer	2.02 × 10^−5^
Rap1 signaling pathway	3.30 × 10^−5^
Lysine degradation	3.33 × 10^−5^
Glioma	6.23 × 10^−5^
Signaling pathways regulating pluripotency of stem cells	9.51 × 10^−5^
EVs: MDS vs. CTR	
ECM-receptor interaction	1.16 × 10^−26^
Fatty acid biosynthesis	1.41 × 10^−8^
ErbB signaling pathway	1.41 × 10^−8^
Proteoglycans in cancer	1.57 × 10^−8^
Axon guidance	3.54 × 10^−8^
Glioma	5.42 × 10^−8^
Mucin type O-Glycan biosynthesis	5.18 × 10^−6^
Estrogen signaling pathway	3.52 × 10^−5^
Focal adhesion	5.71 × 10^−5^
Signaling pathways regulating pluripotency of stem cells	5.71 × 10^−5^
plasma: early vs. advanced MDS	
Amphetamine addiction	1.56 × 10^−7^
Signaling pathways regulating pluripotency of stem cells	5.60 × 10^−6^
Transcriptional misregulation in cancer	1.35 × 10^−5^
Gap junction	2.31 × 10^−5^
Glioma	3.38 × 10^−5^
FoxO signaling pathway	5.34 × 10^−5^
Hippo signaling pathway	1.16 × 10^−4^
ErbB signaling pathway	1.72 × 10^−4^
Proteoglycans in cancer	1.75 × 10^−4^
TGF-beta signaling pathway	2.54 × 10^−4^
EVs: early vs. advanced MDS	
Biotin metabolism	7.16 × 10^−3^
Central carbon metabolism in cancer	7.16 × 10^−3^
Signaling pathways regulating pluripotency of stem cells	7.16 × 10^−3^
Lysine degradation	9.83 × 10^−3^
TGF-beta signaling pathway	9.83 × 10^−3^
Steroid biosynthesis	1.29 × 10^−2^
Glioma	1.39 × 10^−2^
RNA transport	1.41 × 10^−2^
ErbB signaling pathway	1.45 × 10^−2^
Morphine addiction	1.45 × 10^−2^

**Table 2:** SncRNAs associated with overall survival of MDS patients. Prediction model coefficients are applicable to the formula of survival risk score. The survival risk score of a total plasma sample = −0.631 × log_2_ (level of miR-1260b) − 0.24 × log_2_ (level of miR-328-3p) + 6.861. Similarly, the survival risk score of an EV sample = 0.615 × log_2_ (level of miR-1237-3p) + 0.917 × log_2_ (level of U33) − 0.106 × log_2_ (level of hsa_piR_019420) − 1.01 × log_2_ (level of miR-548av-5p) − 4.948. A sample is predicted as high (low) risk if its prognostic index is >0 (≤0).

sncRNA	Univariate Cox Regression Analysis			Prediction Model	
	Univariate Cox Regression, *p*-Value	Permutation, *p*-Value	Hazard Ratio	Coefficient	Cross-Validation, *p*-Value
Total plasma					
miR-1260b	0.0007	6 × 10^−4^	0.441	−0.631	0.0002
miR-3191-3p	0.0009	9 × 10^−4^	0.338	n.a.	n.s.
miR-328-3p	0.0009	8 × 10^−4^	0.474	−0.24	0.0008
EV fraction					
miR-1237-3p	2 × 10^−5^	<1 × 10^−7^	20.135	0.615	5 × 10^−7^
U33	0.0006	6 × 10^−4^	2.499	0.917	0.0002
hsa_piR_019420	0.001	<1 × 10^−7^	20.135	−0.106	0.0008
miR-548av-5p	0.001	0.001	0.217	−1.01	0.0009

n.a.—not applicable, n.s.—nonsignificant.

**Table 3:** Cox multivariate analysis for overall survival of MDS patients.

Variable	HR	95.0% CI for HR		*p*
		Lower	Upper	
Age	1.044	0.945	1.153	0.397
Blasts	0.882	0.767	1.013	0.076
Hemoglobin	1.001	0.961	1.043	0.948
Neutrophils	0.778	0.577	1.048	0.099
Platelets	1.003	0.993	1.013	0.602
IPSS-R score	1.410	0.840	2.366	0.193
Combined score (total plasma)	1.764	0.666	4.677	0.254
Combined score (EVs)	5.866	2.262	15.210	<0.001

## Supplementary Materials

The following are available online at https://www.mdpi.com/2073-4409/9/4/794/s1.
