# Supplementary material for: Circulating Small Noncoding RNAs Have Specific Expression Patterns in Plasma and Extracellular Vesicles in Myelodysplastic Syndromes and Are Predictive of Patient Outcome
Source: Cells. 2020 Mar 26;9(4):794. doi: 10.3390/cells9040794 (PMC7226126; doi:10.3390/cells9040794)
Supplement: Supplementary file 1 [file cells-09-00794-s001.pdf]

Circulating small noncoding RNAs have specific patterns in plasma and extracellular vesicles in myelodysplastic syndromes and are predictive of patient outcome

Andrea Hrustincova, Zdenek Krejcik, David Kundrat, Katarina Szikszai, Monika Belickova, Pavla Pecherkova, Jiri Klema, Jitka Vesela, Monika Hruba, Jaroslav Cermak, Tereza Hrdinova, Matyas Krijt, Jan Valka, Anna Jonasova, Michaela Dostalova Merkerova

SUPPLEMENTARY INFORMATION

SUPPLEMENTARY TABLES AND FIGURES

SI 1. Characteristics of the sequencing cohort. The data are presented as the mean and range for all continuous variables. n.a. - not analyzed.

**MDS**	**31**
Age	67 (29–87)
Sex (male/female)	20/11
Diagnosis<br>MLD/RS/5q-/EB1/EB2	 5/5/3/5/13
IPSS-R category<br>very low/low/intermediate/high/very high/n.a.	 3/10/4/6/6/2
Bone marrow blasts (%)	7.4 (0.4–19.4)
White blood count (x10^9/L)	4.3 (1.2-11.9)
Hemoglobin (g/L)	100 (72–138)
Neutrophils (x10^9/L)	2.4 (0.1–8.6)
Platelets (x10^9/L)	136 (13–390)
Cytogenetic features<br>normal karyotype<br>isolated del(5q)<br>complex<br>other<br>n.a.	 13<br>3<br>4<br>8<br>3
IPSS-R karyotype<br>very good/good/intermediate/poor/very poor/n.a.	 0/18/4/2/4/3
Somatic mutations<br>No. of analyzed patients<br>No. of mutations per patient: 0/1/2/3/4/5<br>The most frequent mutations<br>SF3B1/DNMT3A/RUNX1/ASXL1/EZH2/SETBP1	 18 (58%)<br>3/5/6/2/1/1<br><br>5/5/4/3/3/3
Follow-up<br>mean follow-up (months)	 22 (1-61)
Deceased, number of patients<br>mean time to death (months)	19 (61%)<br>19 (1-43)
Follow-up treatment with AZA, number of patients<br>No. of responders<br>No. of patients with stable disease<br>No. of patients with progressive disease	24<br>9<br>6<br>9
**AML-MRC**	**11**
Age	69 (58–77)
Sex (male/female)	6/5
Bone marrow blasts (%)	26.0 (20.1–41.0)
White blood count (x10^9/L)	3.0 (0.8-8.1)
Hemoglobin (g/L)	95 (77–127)
Neutrophils (x10^9/L)	1.0 (0.1–3.4)
Platelets (x10^9/L)	116 (24–258)
Cytogenetic features<br>normal karyotype<br>isolated del(5q)<br>complex	 5<br>1<br>1

other<br>n.a.	2<br>2
**Healthy donors**	**17**
Age	62 (51–73)
Sex (male/female)	8/9

SI 2. Characterization of somatic mutations detected in MDS patients. VAF - variant allele frequency, n.d. – not detected, NA – not available.

ID	Dg	Gene	VAF	DNA change	Protein change	ID (dbSNP, COSMIC)
**683**	EB2	*JAK2*	50	c.1849G>T	p.Val617Phe	rs147001633
		DNMT3A	39	c.2645G>A	p.Arg882His	rs77375493
**775**	EB2	*SF3B1*	50	c.1774G>A	p.Glu592Lys	COSM132936
		RUNX1	22	c.790G>A	p.Gln264Ter	NA
		STAG2	14	c.571_572delAT	p.Ile191Ter	NA
		EZH2	13	c.1310C>T	p.Trp437Ter	NA
**807**	RS-SLD	n.d.	-	-	-	-
**820**	MLD	*SETBP1*	30	c.2602G>A	p.Asp868Asn	rs267607042
		SRSF2	28	c.284C>G	p.Pro95Arg	rs751713049
**824**	EB1	*MLL*	12	c.173_232delCGGCTGTGGCGGCCGCGGCGGCGGCGGCGGGAAGCAGCGGGGCTGGGGTTCCAGGGGGAG	p.Val60_Ala79del	NA
**865**	MLD	*DNMT3A*	34	c.1120C>T	p.Gln374Ter	rs369109129
**985**	RS-MLD	*SF3B1*	42	c.2098A>G	p.Lys700Glu	rs559063155
		ASXL1	32	c.1934dup	p.Gly646TrpfsTer12	rs756958159
**1028**	MLD	*ASXL1*	5	c.2757dup	p.Pro920ThrfsTer4	rs771822198
**1152**	RS-MLD	*DNMT3A*	47	c.2645G>A	p.Arg882His	rs147001633
		SF3B1	45	c.1874G>T	p.Arg625Leu	COSM110695
**1333**	EB1	*DNMT3A*	26	c.2266G>A	p.Glu756Lys	rs1418213272
		SETBP1	24	c.2602G>A	p.Asp868Asn	rs267607042
**1422**	MLD	*SRSF2*	50	c.284C>T	p.Pro95Leu	rs751713049
		SETBP1	50	c.2602G>A	p.Asp868Asn	rs267607042
		RUNX1	49	c.482T>C	p.Leu161Pro	COSM444417
**1511**	5q-	n.d.	-	-	-	-
**1517**	EB2	*ZRSR2*	48	c.1314_1315insAGCCGG	p.R448_R449insSerArg	COSM5762985
		CUX1	38	c.1636A>C	p.Lys546Gln	rs118010189
**1528**	5q-	n.d.	-	-	-	-
**1573**	RS-SLD	*SF3B1*	26	c.2098A>G	p.Lys700Glu	rs559063155
**1592**	EB2	*ASXL1*	50	c.4189G>A	p.Gly1397Ser	COSM133033
		EZH2	49	c.862G>A	p.Arg288Ter	COSM1000721
		TET2	45	c.3852_3854delCTT	p.Phe1285del	COSM2270963
		RUNX1	45	c.1158_1159insG	p.Ser388fsTer212	NA
		PHF6	45	c.385C>T	p.Arg129Ter	COSM4606367

**1803**	EB2	*DNMT3A*	41	c.1728delT	p.Lys577ArgfsTer74	NA
**1834**	EB1	*SF3B1*	37	c.1998G>C	p.Lys666Asn	rs377023736
		RUNX1	35	c.278A>T	p.Asp93Val	NA
		EZH2	7	c.875A>G	p.Tyr292Cys	NA

SI 3. Overall survival of MDS patients in the sequencing cohort stratified according to clinical variables. *p < 0.05, **p < 0.01.

Variable	Cut-off	No. of patients	Median OS (mo)	Hazard ratio (A/B)	95% confidence interval	p value
**Age**	A: < 70 years	17	29.9	1.486	0.595 to 3.711	0.418
	B: ≥ 70 years	14	30.4			
**Gender**	A: female	11	37.8	0.669	0.268 to 1.669	0.393
	B: male	20	23.5			
**Diagnosis**	A: early MDS	13	36.3	0.414	0.162 to 1.054	0.038 *
	B: advanced MDS	18	20.1			
**IPSS-R category**	A: (very) low	17	36.3	0.303	0.108 to 0.853	0.006 **
	B: (very) high	12	16			
**IPSS-R karyotype**	A: (very) good	18	29.9	0.409	0.140 to 1.195	0.046 *
	B: int/(very) poor	10	20.1			
**Bone marrow blasts**	A: < 5%	14	36.3	0.381	0.148 to 0.981	0.024 *
	B: ≥ 5%	17	20.1			
**Hemoglobin**	A: < 100 g/L	18	20.6	2.065	0.837 to 5.095	0.108
	B: ≥ 100 g/L	13	30.7			
**Neutrophils**	A: < $0.8x10^9$/L	8	13.7	2.694	0.755 to 9.619	0.034 *
	B: ≥ $0.8x10^9$/L	23	30.7			
**Platelets**	A: < $100x10^9$/L	17	23.5	1.832	0.739 to 4.545	0.180
	B: ≥ $100x10^9$/L	14	37.8			

SI 4. Characteristics of the validation cohort analyzed with ddPCR. The data are presented as the mean and range for all continuous variables. n.a. - not analyzed.

**MDS**	**36**
Age	60 (29–81)
Sex (male/female)	26/10
Diagnosis<br>SLD/MLD/RS/5q-/EB1/EB2	 2/10/2/1/5/16
IPSS-R category<br>very low/low/intermediate/high/very high/n.a.	 6/10/5/4/11
Bone marrow blasts (%)	7.1 (0.2–19.8)
White blood count (x10^9/L)	3.8 (0.9-11.4)
Hemoglobin (g/L)	101 (59–138)
Neutrophils (x10^9/L)	1.8 (0.1–7.6)
Platelets (x10^9/L)	126 (26–273)
Cytogenetic features<br>normal karyotype<br>isolated del(5q)<br>complex<br>other	 16<br>2<br>9<br>9
IPSS-R karyotype<br>very good/good/intermediate/poor/very poor	 1/20/3/3/9
Follow-up<br>mean follow-up (months)	 11 (0-77)
Deceased, number of patients<br>mean time to death (months)	17 (47%)<br>7 (1-23)
**AML-MRC**	**7**
Age	68 (62–76)
Sex (male/female)	5/2
Bone marrow blasts (%)	36.3 (23.0–62.0)
White blood count (x10^9/L)	2.2 (1.0-4.8)
Hemoglobin (g/L)	100 (85–127)
Neutrophils (x10^9/L)	0.8 (0.1–2.9)
Platelets (x10^9/L)	91 (10–202)
Cytogenetic features<br>normal karyotype<br>complex<br>other	 2<br>3<br>2
**Healthy donors**	**12**
Age	69 (58–77)
Sex (male/female)	4/8

SI 5. Characterization of the extracellular particles in plasma. (A) Particle size (mean size with 10th and 90th percentiles) measured by nanoparticle tracking analysis (NTA). (B) NTA report of representative samples of total plasma from one healthy control and one MDS-EB2 patient. (C) RNA yield obtained from (a) 1 ml of total plasma and (b) EV fractions that were isolated from 1 ml of the plasma is plotted.

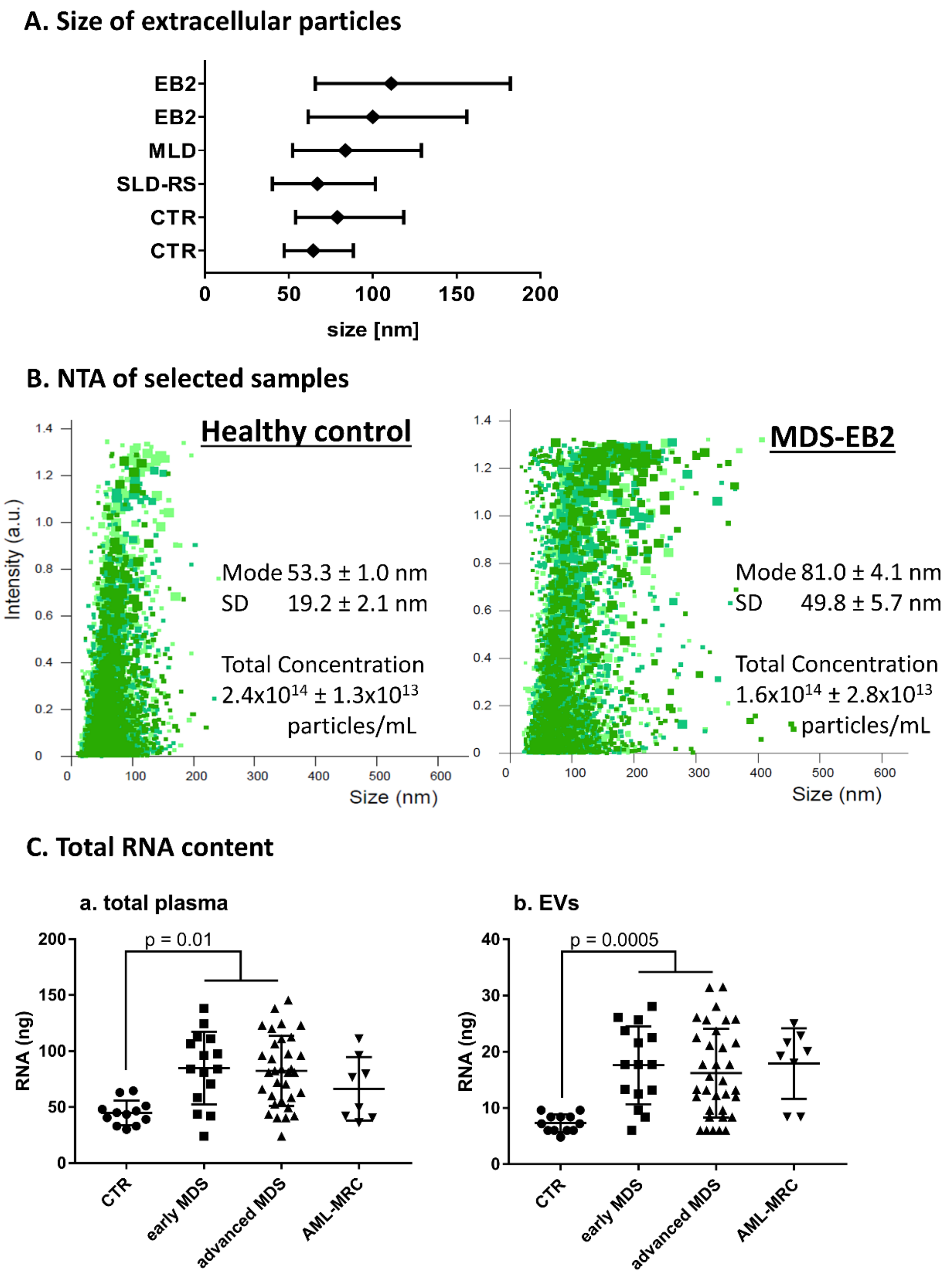

SI 6. Transmission electron microscopy (TEM) (A) and nanoparticle tracking analysis (NTA) (B) of one representative sample of the EV fraction. Characterization of exosomes in EV samples by western blotting (C). Common exosome markers CD9, CD81, and cell organelle marker calnexin (negative control). MDS – MDS samples, CTR – control samples, TCL – K562 Total Cell Lysate was loaded as a positive or negative control.

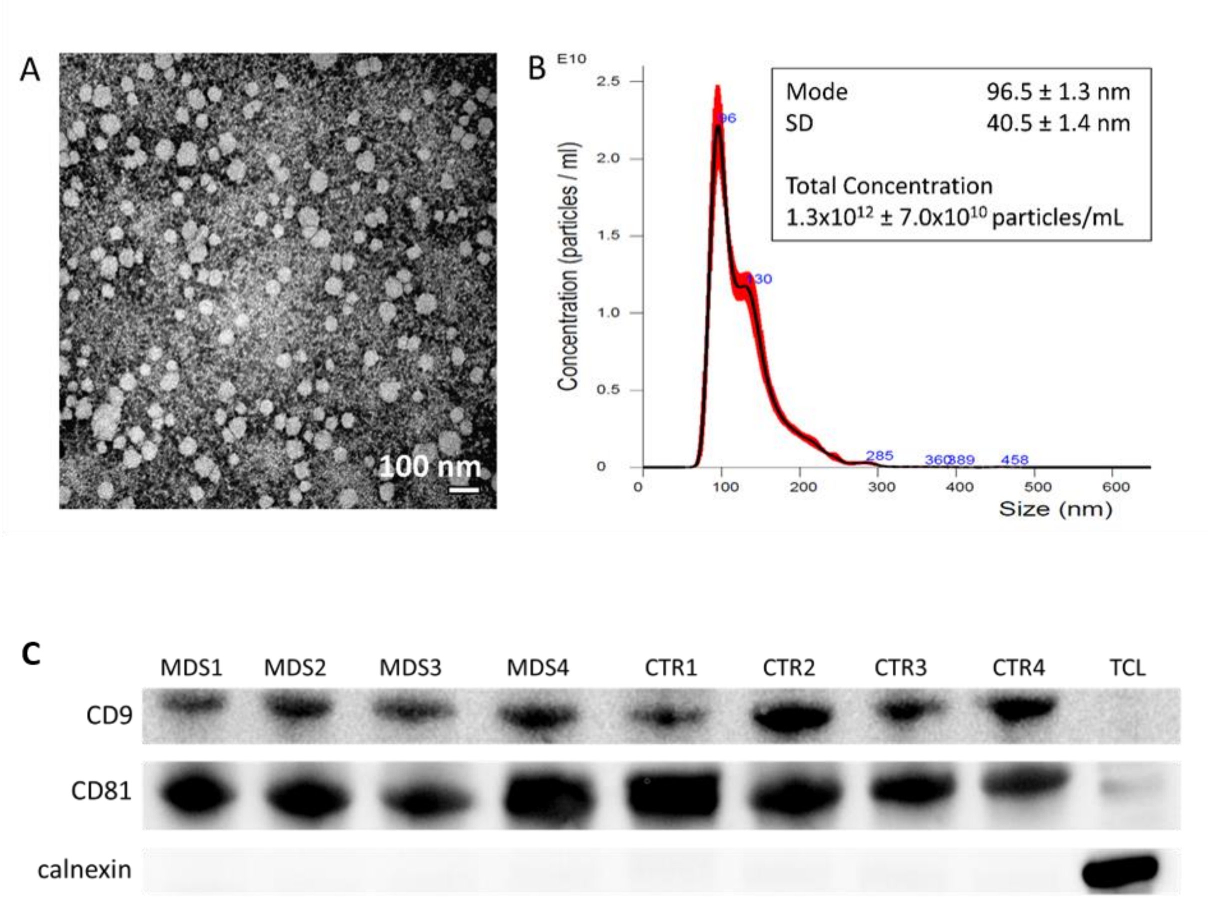

SI 7. Length of reads in relation to mapping rate.

**Read length**	**Proportion of reads mapped to human genome**
15-19 bp	63%
20-22 bp	100%
23-30 bp	93%
31-70 bp	23%
71-85 bp	5%

SI 8. Taxonomy profile of reads mappable to nonhuman genomes. The proportion of the reads assigned to individual phyla is shown.

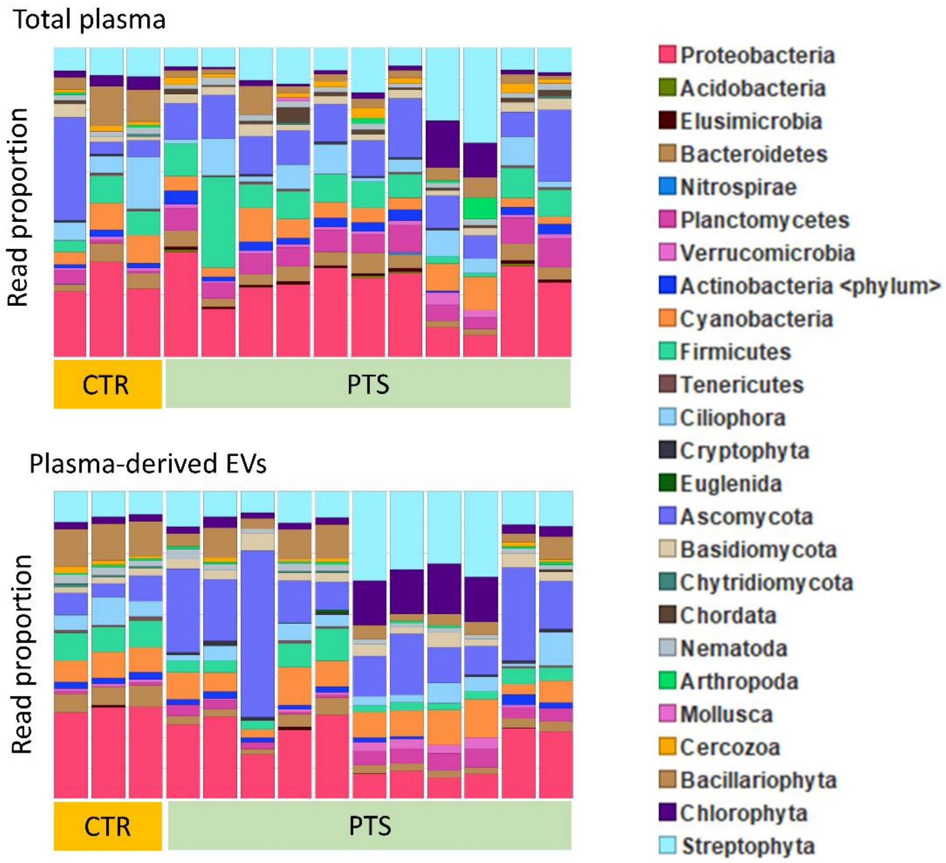

SI 9. Differentially expressed sncRNAs between paired samples of total plasma and EV fractions from MDS patients. Of the 419 significant sncRNAs (|logFC| > 1 and q < 0.05), only the top 50 genes are listed. logCPM – binary logarithm of counts per million, logFC – binary logarithm of fold change, q-value – False Discovery Rate (FDR) adjusted p-value.

gene	logCPM_PL	logCPM_EV	logFC	p-value	q-value
hsa_piR_001170/gb/DQ571526/Homo	5.24	0.25	6.76	9.64E-19	2.81E-15
hsa_piR_020009/gb/DQ597484/Homo	6.05	0.74	6.39	7.71E-17	1.12E-13
Homo_sapiens_chr2.trna19-GlyGCC	7.95	5.28	2.55	7.94E-16	7.70E-13
hsa-miR-99b-5p	11.07	8.70	2.25	1.70E-15	1.24E-12
Homo_sapiens_chr16.trna25-GlyGCC	10.20	7.23	2.78	2.64E-15	1.54E-12
Homo_sapiens_chr1.trna68-GlyGCC	8.35	5.79	2.41	4.92E-15	2.05E-12
Homo_sapiens_chr16.trna24-GlyGCC	6.94	4.45	2.48	4.81E-15	2.05E-12
Homo_sapiens_chr16.trna34-GlyCCC	8.64	6.49	2.08	5.77E-14	2.10E-11
Homo_sapiens_chr1.trna74-GluCTC	7.67	5.56	1.98	2.24E-13	7.24E-11
hsa-miR-191-5p	14.92	11.94	2.75	2.67E-13	7.52E-11
Homo_sapiens_chr1.trna37-GlyGCC	6.83	4.57	2.15	2.84E-13	7.52E-11
Ro-associated	15.39	12.81	2.64	3.22E-13	7.81E-11
hsa-miR-125a-5p	13.37	11.57	1.87	3.91E-13	8.75E-11
hsa-miR-103b	11.99	9.33	2.42	4.22E-13	8.77E-11
Homo_sapiens_chr1.trna39-GlyGCC	7.42	5.32	1.93	4.81E-13	9.33E-11
Homo_sapiens_chr1.trna98-ValCAC	7.93	5.08	2.72	9.01E-13	1.64E-10
hsa-miR-1301-3p	7.84	5.84	1.73	9.65E-13	1.65E-10
Homo_sapiens_chr1.trna116-GluCTC	9.89	8.12	1.79	1.82E-12	2.94E-10
Homo_sapiens_chr16.trna19-GlyGCC	9.19	6.48	2.47	1.94E-12	2.97E-10
hsa-miR-3135b	8.01	6.01	1.77	2.34E-12	3.25E-10
hsa_piR_016658/gb/DQ592931/Homo	12.31	9.57	2.71	2.33E-12	3.25E-10
hsa-miR-744-5p	11.75	9.79	1.88	2.69E-12	3.55E-10
U95	7.46	5.22	2.08	3.50E-12	4.42E-10
hsa-miR-941	8.53	6.94	1.59	4.09E-12	4.96E-10
Homo_sapiens_chr1.trna77-GluCTC	7.66	5.92	1.65	4.44E-12	4.97E-10
5S	10.20	8.11	1.96	4.39E-12	4.97E-10
Homo_sapiens_chr15.trna11-GluTTC	8.20	5.88	2.23	5.05E-12	5.07E-10
Homo_sapiens_chr5.trna15-ValAAC	8.90	5.98	2.55	4.96E-12	5.07E-10
Homo_sapiens_chr6.trna77-GluCTC	7.50	5.62	1.77	4.77E-12	5.07E-10
Homo_sapiens_chr5.trna18-ValCAC	7.83	4.94	2.61	5.62E-12	5.46E-10
Homo_sapiens_chr17.trna5-GlyGCC	8.19	6.07	2.06	5.96E-12	5.60E-10
hsa-miR-103a-3p	13.72	11.01	2.43	7.69E-12	6.40E-10
hsa-miR-127-3p	11.96	9.75	1.99	7.49E-12	6.40E-10
Homo_sapiens_chr6.trna152-ValCAC	8.85	5.55	2.55	7.60E-12	6.40E-10
Homo_sapiens_chr6.trna87-GluCTC	7.24	5.22	1.93	7.37E-12	6.40E-10
Homo_sapiens_chr1.trna59-GluCTC	7.20	4.92	2.16	1.11E-11	8.98E-10
Homo_sapiens_chr2.trna27-GlyCCC	7.86	5.92	1.75	1.45E-11	1.14E-09
hsa_piR_008114/gb/DQ581033/Homo	9.08	7.42	1.93	1.49E-11	1.14E-09

Homo_sapiens_chr21.trna2-GlyGCC	7.93	5.55	2.14	1.59E-11	1.19E-09
Homo_sapiens_chr1.trna41-GlyGCC	8.09	6.13	1.88	2.27E-11	1.65E-09
hsa-miR-671-3p	7.37	5.77	1.42	2.36E-11	1.68E-09
Homo_sapiens_chr1.trna71-GluCTC	9.86	7.93	1.78	2.50E-11	1.73E-09
hsa-miR-409-3p	12.01	10.56	1.62	2.73E-11	1.85E-09
Homo_sapiens_chr13.trna3-GluTTC	8.37	6.37	1.93	3.68E-11	2.43E-09
hsa_piR_016659/gb/DQ592932/Homo	10.83	8.88	2.01	4.13E-11	2.67E-09
hsa-miR-342-3p	13.09	11.24	1.72	5.12E-11	3.24E-09
Homo_sapiens_chr5.trna6-ValCAC	5.75	4.16	1.70	5.53E-11	3.42E-09
hsa_piR_017716/gb/DQ594453/Homo	7.86	5.24	2.17	1.04E-10	6.32E-09
Homo_sapiens_chr15.trna2-LysCTT	8.34	6.72	1.57	1.07E-10	6.33E-09
Homo_sapiens_chr1.trna80-GluCTC	9.76	7.48	1.98	1.20E-10	7.01E-09

SI 10. Differentially expressed sncRNAs between paired samples of total plasma and EV fractions from healthy controls (|logFC| > 1 and q < 0.05). logCPM – binary logarithm of counts per million, logFC – binary logarithm of fold change, q-value – False FDR adjusted p-value.

gene	logCPM_PL	logCPM_EV	logFC	p-value	q-value
hsa_piR_020326/gb/DQ597916/Homo	6.20	2.62	3.10	1.37E-07	3.84E-04
de novo miRNA_chr1:11968900..11968941:-	6.34	2.15	4.51	4.94E-07	6.95E-04
hsa-miR-3649	5.26	6.45	-1.49	1.48E-06	1.22E-03
hsa_piR_000765/gb/DQ570956/Homo	7.91	5.43	2.22	2.17E-06	1.22E-03
hsa_piR_016659/gb/DQ592932/Homo	10.48	9.08	1.28	2.02E-06	1.22E-03
Homo_sapiens_chr1.trna80-GluCTC	8.30	6.04	1.90	2.90E-06	1.36E-03
hsa-miR-324-5p	8.21	6.72	1.33	5.63E-06	2.26E-03
hsa-miR-191-5p	13.63	11.19	1.77	8.41E-06	2.72E-03
Homo_sapiens_chr1.trna4-GlyCCC	7.68	4.67	2.15	8.71E-06	2.72E-03
hsa-miR-643	3.52	4.90	-1.28	1.02E-05	2.86E-03
hsa-miR-4508	5.35	4.23	1.15	1.14E-05	2.93E-03
de novo miRNA_chr17:79320004..79320044:-	7.96	3.90	4.82	1.73E-05	3.48E-03
hsa_piR_016658/gb/DQ592931/Homo	10.37	8.99	1.40	1.69E-05	3.48E-03
Homo_sapiens_chr1.trna71-GluCTC	8.68	7.03	1.46	1.59E-05	3.48E-03
Homo_sapiens_chr16.trna19-GlyGCC	7.52	5.68	1.42	3.10E-05	5.41E-03
Homo_sapiens_chr6.trna77-GluCTC	6.03	4.55	1.24	3.27E-05	5.41E-03
hsa_piR_017716/gb/DQ594453/Homo	6.66	4.36	1.84	4.55E-05	6.39E-03
Homo_sapiens_chr16.trna24-GlyGCC	5.24	3.91	1.36	4.47E-05	6.39E-03
Ro-associated	13.99	12.62	1.49	4.20E-05	6.39E-03
hsa-miR-548v	3.57	4.79	-1.17	6.40E-05	8.18E-03
hsa-miR-103b	10.70	8.99	1.63	8.85E-05	9.21E-03
hsa-miR-203a-3p	6.99	8.39	-1.60	8.18E-05	9.21E-03
Homo_sapiens_chr1.trna68-GlyGCC	6.29	4.99	1.10	8.71E-05	9.21E-03
5S	9.15	7.99	1.10	8.31E-05	9.21E-03
de novo miRNA_chr5:176932110..176932198:-	4.24	-0.31	4.79	1.05E-04	1.00E-02
Homo_sapiens_chr1.trna77-GluCTC	6.43	5.02	1.18	1.07E-04	1.00E-02
Homo_sapiens_chr16.trna34-GlyCCC	6.58	5.45	1.11	1.05E-04	1.00E-02
Homo_sapiens_chr1.trna74-GluCTC	5.99	4.79	1.07	1.54E-04	1.38E-02
Homo_sapiens_chr6.trna87-GluCTC	5.98	4.71	1.17	1.57E-04	1.38E-02
hsa-miR-744-5p	10.35	9.12	1.10	1.63E-04	1.39E-02
de novo miRNA_chr7:143079637..143079686:+	5.77	3.72	2.03	1.77E-04	1.47E-02
Homo_sapiens_chr16.trna25-GlyGCC	7.66	6.07	1.26	2.29E-04	1.84E-02
Homo_sapiens_chr15.trna2-LysCTT	5.62	4.47	1.09	2.81E-04	2.19E-02
de novo miRNA_chr22:20052702..20052764:+	4.29	0.50	4.17	2.90E-04	2.20E-02
de novo miRNA_chr2:180934649..180934696:+	3.97	7.72	-6.27	3.19E-04	2.36E-02
Homo_sapiens_chr1.trna133-GlyCCC	4.10	2.00	2.09	3.79E-04	2.73E-02
hsa-miR-454-3p	7.83	5.41	1.83	4.22E-04	2.97E-02
de novo miRNA_chr9:127107293..127107341:-	6.15	1.24	6.11	5.02E-04	3.28E-02
Homo_sapiens_chr1.trna59-GluCTC	5.67	4.40	1.13	4.94E-04	3.28E-02

hsa-miR-103a-3p	12.22	10.47	1.53	6.61E-04	3.95E-02
de novo miRNA_chr6:26305715..26305784:-	6.85	3.77	5.92	6.50E-04	3.95E-02
hsa-miR-203b-5p	5.43	6.46	-1.06	7.84E-04	4.59E-02
Homo_sapiens_chr21.trna2-GlyGCC	5.97	4.44	1.10	8.65E-04	4.77E-02
Homo_sapiens_chr6.trna9-ValCAC	4.63	3.53	1.01	8.39E-04	4.77E-02

SI 11. Differentially expressed sncRNAs in total plasma samples from MDS patients *vs.* healthy controls. Of the 391 significant sncRNAs (|logFC| > 1 and q < 0.05), only the top 50 genes are listed. logCPM – binary logarithm of counts per million, logFC – binary logarithm of fold change, q-value – FDR adjusted p-value.

gene	logCPM_MDS	logCPM_CTR	logFC	p-value	q-value
Homo_sapiens_chr6.trna152-ValCAC	8.77	5.13	3.71	7.33E-14	2.48E-10
Homo_sapiens_chr15.trna11-GluTTC	8.13	5.75	2.43	1.84E-12	3.11E-09
hsa-miR-4784	7.22	5.22	2.04	4.82E-12	5.44E-09
Homo_sapiens_chr1.trna62-LysTTT	6.76	4.17	2.64	1.44E-10	8.13E-08
Homo_sapiens_chr1.trna98-ValCAC	7.84	5.12	2.82	1.30E-10	8.13E-08
Homo_sapiens_chr5.trna15-ValAAC	8.81	6.28	2.59	1.36E-10	8.13E-08
Homo_sapiens_chr2.trna27-GlyCCC	7.78	5.92	1.90	1.85E-10	8.97E-08
hsa-miR-3135b	7.93	5.72	2.23	1.68E-09	7.11E-07
Homo_sapiens_chr1.trna41-GlyGCC	8.02	5.73	2.34	2.34E-09	8.82E-07
Homo_sapiens_chr16.trna25-GlyGCC	10.12	7.75	2.38	2.89E-09	8.99E-07
Homo_sapiens_chr16.trna34-GlyCCC	8.57	6.70	1.90	2.92E-09	8.99E-07
Homo_sapiens_chr5.trna18-ValCAC	7.74	5.20	2.64	3.87E-09	1.09E-06
Homo_sapiens_chr16.trna29-ProAGG	4.70	1.89	3.10	4.56E-09	1.19E-06
Homo_sapiens_chr2.trna19-GlyGCC	7.87	5.71	2.21	5.40E-09	1.31E-06
hsa_piR_020496/gb/DQ598175/Homo	4.90	2.30	2.84	5.82E-09	1.31E-06
hsa-miR-34a-5p	8.57	6.39	2.19	6.89E-09	1.43E-06
de novo miRNA_chr10:105315004..105315059:-	0.00	4.69	-7.28	7.17E-09	1.43E-06
hsa-miR-150-5p	13.36	10.67	2.69	9.47E-09	1.69E-06
hsa-miR-99b-3p	7.37	4.98	2.44	9.36E-09	1.69E-06
Homo_sapiens_chr18.trna4-LysCTT	8.35	5.24	3.14	1.75E-08	2.96E-06
hsa-miR-615-5p	3.80	4.87	-1.13	2.21E-08	3.40E-06
U104	10.16	7.16	3.01	2.14E-08	3.40E-06
hsa-miR-99b-5p	11.01	8.37	2.65	3.12E-08	4.33E-06
hsa_piR_001170/gb/DQ571526/Homo	5.16	1.39	4.85	3.32E-08	4.33E-06
Homo_sapiens_chr1.trna119-LysCTT	6.43	4.25	2.30	3.28E-08	4.33E-06
U95	7.37	4.22	3.23	2.97E-08	4.33E-06
Homo_sapiens_chr15.trna2-LysCTT	8.26	5.74	2.56	4.39E-08	5.51E-06
hsa-miR-206	5.70	7.24	-1.58	5.06E-08	6.12E-06
Homo_sapiens_chr1.trna37-GlyGCC	6.76	4.62	2.19	6.04E-08	7.06E-06
Homo_sapiens_chr1.trna39-GlyGCC	7.34	5.12	2.26	6.74E-08	7.61E-06
U50B	6.20	2.54	3.86	9.36E-08	1.02E-05
U31	6.94	3.74	3.33	1.09E-07	1.15E-05
Homo_sapiens_chr1.trna68-GlyGCC	8.27	6.39	1.90	1.50E-07	1.52E-05
Homo_sapiens_chr19.trna2-GlyTCC	5.14	2.73	2.66	1.53E-07	1.52E-05
Homo_sapiens_chr16.trna10-LysCTT	6.84	4.48	2.44	1.59E-07	1.54E-05
hsa-miR-6126	6.75	5.08	1.70	1.74E-07	1.64E-05
de novo miRNA_chr17:42344334..42344386:+	0.00	5.92	-8.56	1.86E-07	1.68E-05
Homo_sapiens_chr19.trna13-ValCAC	8.61	6.96	1.69	1.88E-07	1.68E-05
de novo miRNA_chr17:79320004..79320044:-	2.31	8.05	-5.98	2.12E-07	1.84E-05

hsa-miR-4732-5p	7.26	8.74	-1.48	2.27E-07	1.92E-05
U63	5.64	2.06	3.94	2.38E-07	1.96E-05
Homo_sapiens_chr14.trna13-LysCTT	7.74	5.46	2.33	2.73E-07	2.15E-05
Homo_sapiens_chr2.trna20-GluTTC	5.07	3.03	2.16	2.72E-07	2.15E-05
Homo_sapiens_chr11.trna5-LysTTT	5.68	3.76	2.00	3.16E-07	2.43E-05
Homo_sapiens_chr17.trna2-LysTTT	4.87	3.08	1.94	3.94E-07	2.97E-05
de novo miRNA_chr16:89206351..89206427:+	0.00	6.57	-9.23	4.26E-07	3.14E-05
Homo_sapiens_chr16.trna9-ProAGG	6.81	4.48	2.43	4.56E-07	3.26E-05
U20	7.14	4.55	2.68	4.61E-07	3.26E-05
hsa_piR_020009/gb/DQ597484/Homo	5.97	2.72	3.67	4.80E-07	3.32E-05
hsa-miR-491-5p	6.57	5.34	1.20	5.00E-07	3.32E-05

SI 12. Differentially expressed sncRNAs in EV samples from MDS patients *vs.* healthy controls. Of the 219 significant sncRNAs (|logFC| > 1 and q < 0.05), only the top 50 genes are listed. logCPM – binary logarithm of counts per million, logFC – binary logarithm of fold change, q-value – FDR adjusted p-value.

gene	logCPM_MDS	logCPM_CTR	logFC	p-value	q-value
hsa-miR-6786-5p	3.92	5.23	-1.36	1.68E-09	2.85E-06
de novo miRNA_chr17:79320004..79320044:-	0.00	4.11	-7.07	1.61E-09	2.85E-06
de novo miRNA_chr6:26305715..26305784:-	0.00	3.99	-6.95	1.44E-08	9.76E-06
Homo_sapiens_chr18.trna4-LysCTT	7.26	4.14	3.17	1.39E-08	9.76E-06
U31	6.42	2.97	3.57	1.21E-08	9.76E-06
Homo_sapiens_chr11.trna14-LysTTT	5.99	4.06	1.99	1.93E-08	1.09E-05
hsa-miR-4732-5p	6.59	8.18	-1.61	2.34E-08	1.13E-05
Homo_sapiens_chr1.trna54-LysTTT	5.29	3.50	1.81	3.50E-07	1.19E-04
hsa-miR-3194-3p	4.93	6.45	-1.51	7.62E-07	2.13E-04
de novo miRNA_chr22:31284147..31284211:+	0.00	2.97	-5.79	8.16E-07	2.13E-04
U104	9.56	6.97	2.60	7.35E-07	2.13E-04
de novo miRNA_chr2:43417763..43417825:-	0.00	3.30	-6.16	2.27E-06	5.49E-04
hsa-miR-5010-5p	5.43	6.67	-1.23	3.18E-06	7.12E-04
hsa-miR-548v	3.79	4.97	-1.18	3.57E-06	7.12E-04
U22	4.67	1.87	3.20	3.48E-06	7.12E-04
de novo miRNA_chr1:12647947..12648008:-	0.00	2.64	-5.42	4.51E-06	7.64E-04
U20	6.93	4.60	2.38	4.28E-06	7.64E-04
U3	7.32	5.33	2.02	4.41E-06	7.64E-04
Homo_sapiens_chr15.trna2-LysCTT	6.69	4.66	2.06	5.38E-06	8.68E-04
de novo miRNA_chr9:96064497..96064547:+	0.00	3.56	-6.47	5.96E-06	8.79E-04
de novo miRNA_chr9:96064529..96064607:+	0.00	3.56	-6.47	5.96E-06	8.79E-04
U26	8.35	6.12	2.25	6.66E-06	9.03E-04
U50B	4.97	2.08	3.14	6.62E-06	9.03E-04
hsa_piR_020496/gb/DQ598175/Homo	2.99	1.15	2.15	7.03E-06	9.09E-04
U74	6.14	3.43	2.78	7.24E-06	9.09E-04
de novo miRNA_chr10:105315004..105315059:-	0.00	2.20	-4.97	9.97E-06	1.21E-03
hsa-miR-6784-3p	5.05	6.93	-1.89	1.30E-05	1.51E-03
U24	4.77	2.31	2.63	1.79E-05	2.02E-03
U25	4.57	1.96	2.86	2.86E-05	3.13E-03
Homo_sapiens_chr11.trna5-LysTTT	5.02	3.51	1.55	3.52E-05	3.56E-03
Homo_sapiens_chr16.trna29-ProAGG	3.50	1.67	2.10	3.44E-05	3.56E-03
mgU2-19/30	5.22	3.52	1.83	3.57E-05	3.56E-03
hsa-miR-379-5p	9.13	6.70	2.44	3.90E-05	3.77E-03
Homo_sapiens_chr7.trna16-CysGCA	0.93	5.10	-5.04	4.17E-05	3.93E-03
hsa-miR-6891-5p	4.92	5.97	-1.03	4.59E-05	4.10E-03
Homo_sapiens_chr1.trna111-HisGTG	4.67	1.88	3.08	4.54E-05	4.10E-03
Homo_sapiens_chr1.trna36-LeuCAG	5.57	3.74	1.87	4.91E-05	4.16E-03
SNORD119	7.31	5.16	2.17	5.43E-05	4.49E-03
Homo_sapiens_chr16.trna6-ProCGG	5.93	4.01	1.95	5.99E-05	4.62E-03

U33	7.36	5.79	1.59	5.98E-05	4.62E-03
U3-3	6.86	5.24	1.65	5.78E-05	4.62E-03
HBII-85-10	3.65	1.78	2.18	6.27E-05	4.72E-03
hsa-miR-323a-3p	6.47	4.60	1.90	6.58E-05	4.75E-03
mgh28S-2411	5.17	2.87	2.43	6.48E-05	4.75E-03
hsa-miR-34a-5p	8.06	6.62	1.45	7.07E-05	4.83E-03
Homo_sapiens_chr14.trna13-LysCTT	6.38	4.66	1.73	6.98E-05	4.83E-03
U3-4	6.53	4.96	1.59	7.12E-05	4.83E-03
hsa-miR-3944-3p	4.96	3.54	1.44	7.35E-05	4.88E-03
hsa-miR-423-3p	10.04	8.24	1.81	7.54E-05	4.91E-03
de novo miRNA_chr9:120405289..120405371:-	7.57	0.00	10.59	7.82E-05	5.00E-03

SI 13. Numbers of deregulated circulating sncRNAs in (A) MDS patients *vs.* healthy controls, (B) MDS *vs.* AML-MRC patients, and (C) early *vs.* advanced subtypes of MDS. The numbers in the table show counts of significantly deregulated sncRNAs (increased/decreased in the 1st sample group compared to the 2nd sample group); $|logFC| > 1$ and $q < 0.05$) in total plasma and plasma-derived EVs.

**Type of transcripts**	**A MDS *vs.* CTR**		**B MDS *vs.* AML-MRC**		**C early *vs.* adv. MDS**	
	**plasma**	**EVs**	**plasma**	**EVs**	**plasma**	**EVs**
**all annotated RNAs**	**316/75**	**179/40**	**1/8**	**0/14**	**81/19**	**34/9**
**annotated miRNAs**	112/36	65/18	0/3	0/1	55/12	24/7
**de novo identified miRNAs**	19/39	26/20	1/5	0/10	12/4	8/0
**piRNAs**	24/0	15/1	0/0	0/1	7/1	1/2
**tRNAs**	120/0	43/1	0/0	0/2	5/1	1/0
**other annotated RNAs**	41/0	30/0	0/0	0/0	2/1	0/0

SI 14. Numbers of sncRNAs differentially represented (|logFC| > 1 and q < 0.05) between A) MDS and healthy controls and B) early and advanced subtypes of MDS and their overlaps in the two types of tested material (total plasma and plasma-derived EVs).

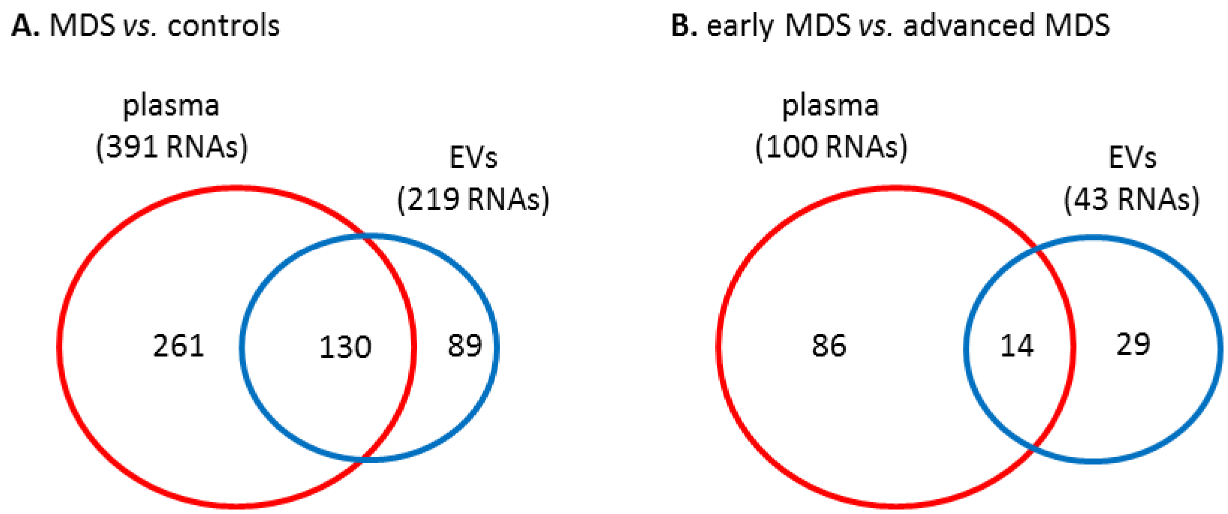

SI 15. Hematopoiesis-related miRNAs with different expression levels between MDS patients and controls. Statistical parameters were computed by the exact test in edgeR (*q < 0.05, **q < 0.01, ***q < 0.001). Total plasma - right parts of the graphs, plasma-derived EVs - left parts of the graphs, CTR - controls, MDS1 - early MDS (MDS-SLD, MDS-MLD, and MDS-5q-), MDS2 - advanced MDS (MDS-EB1 and MDS-EB2), AML - AML-MRC.

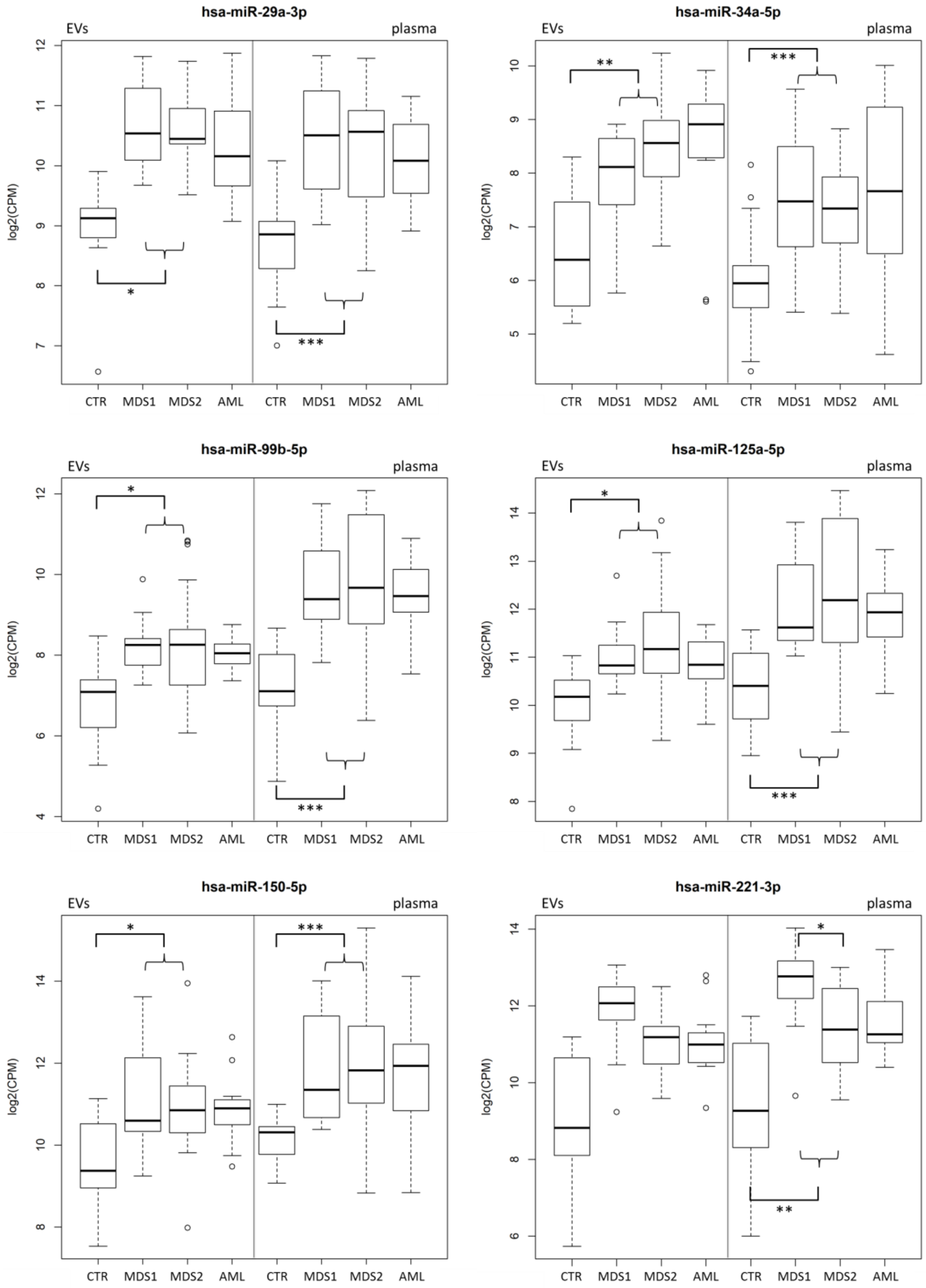

SI 16. Other types of sncRNAs with different expression levels between MDS patients and controls. Statistical parameters were computed by the exact test in edgeR (*q < 0.05, **q < 0.01, ***q < 0.001). Total plasma - right parts of the graphs, plasma-derived EVs - left parts of the graphs, CTR - controls, MDS1 - early MDS (MDS-SLD, MDS-MLD, and MDS-5q-), MDS2 - advanced MDS (MDS-EB1 and MDS-EB2), AML - AML-MRC.

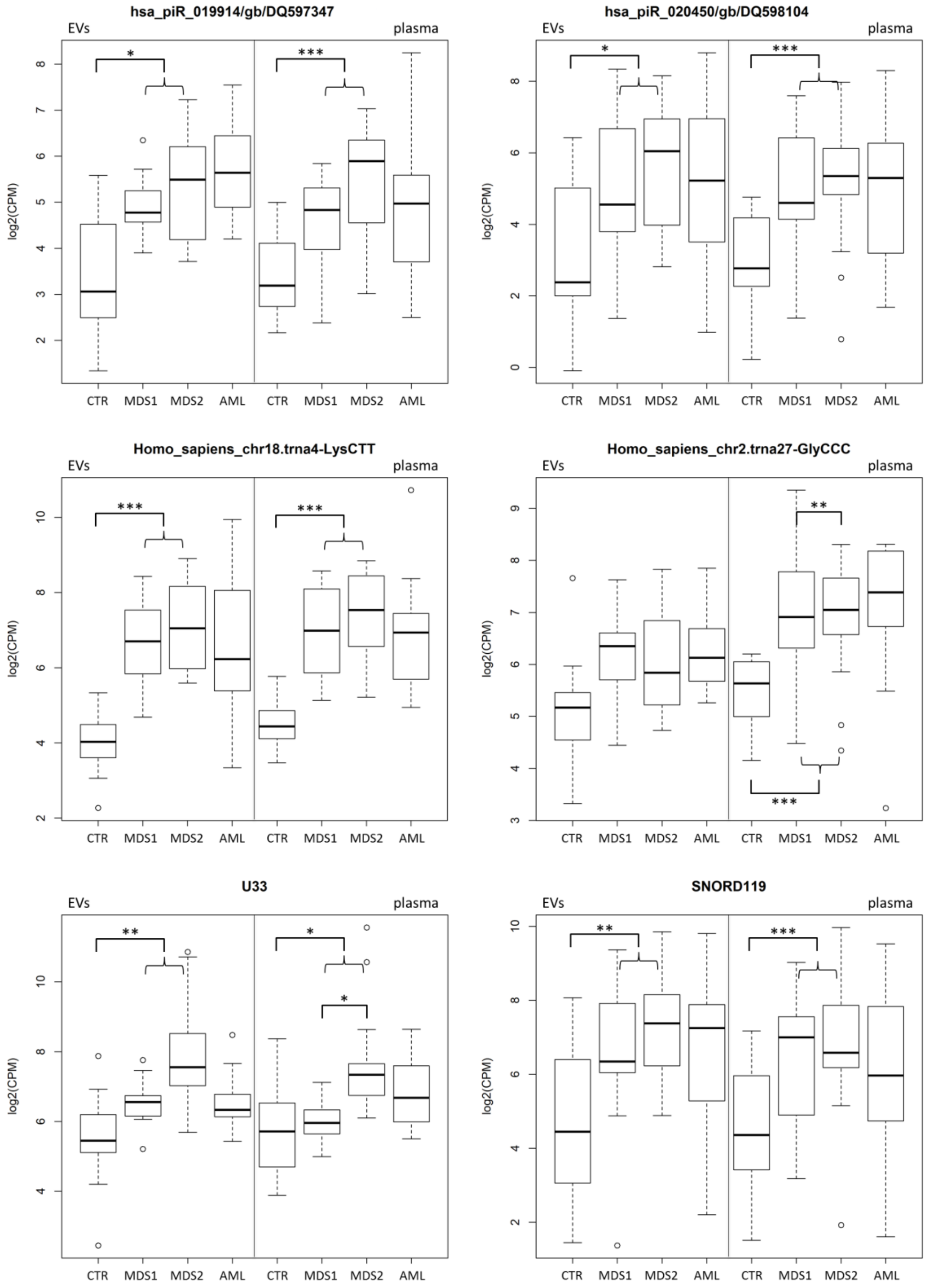

SI 17. Differentially expressed sncRNAs in total plasma samples from MDS *vs.* AML-MRC patients (|logFC| > 1 and q < 0.05). logCPM – binary logarithm of counts per million, logFC – binary logarithm of fold change, q-value – FDR adjusted p-value.

gene	logCPM_MDS	logCPM_AML	logFC	p-value	q-value
hsa-miR-125b-5p	9.97	13.70	-3.73	3.21E-17	1.18E-13
hsa-miR-4324	4.99	7.11	-2.15	3.29E-09	6.07E-06
de novo miRNA_chr3:53118888..53118934:+	6.49	0.00	9.16	4.94E-06	6.07E-03
de novo miRNA_chr15:70482436..70482474:+	0.00	5.09	-7.74	1.48E-05	1.36E-02
hsa-miR-5694	5.17	6.27	-1.15	1.21E-04	4.96E-02
de novo miRNA_chr17:42344334..42344386:+	0.00	3.05	-5.58	7.97E-05	4.96E-02
de novo miRNA_chr22:31284147..31284211:+	0.00	2.00	-4.35	1.15E-04	4.96E-02
de novo miRNA_chr7:74438675..74438732:+	0.00	2.55	-5.01	1.16E-04	4.96E-02
de novo miRNA_chr7:74807675..74807732:-	0.00	2.55	-5.01	1.16E-04	4.96E-02

SI 18. Differentially expressed sncRNAs in EV samples from MDS *vs.* AML-MRC patients (|logFC| > 1 and q < 0.05). logCPM – binary logarithm of counts per million, logFC – binary logarithm of fold change, q-value – FDR adjusted p-value.

**gene**	**logCPM_MDS**	**logCPM_AML**	**logFC**	**p-value**	**q-value**
de novo miRNA_chr13:62820841..62820876:-	0.00	3.06	-6.16	7.80E-06	1.44E-02
de novo miRNA_chr15:100437310..100437377:-	0.00	3.59	-6.74	6.47E-06	1.44E-02
de novo miRNA_chr9:96064497..96064547:+	0.00	3.01	-6.11	1.74E-05	1.60E-02
de novo miRNA_chr9:96064529..96064607:+	0.00	3.01	-6.11	1.74E-05	1.60E-02
hsa-miR-513b-3p	3.51	5.40	-1.94	4.10E-05	2.58E-02
Homo_sapiens_chr1.trna21-HisGTG	3.79	6.34	-2.62	4.20E-05	2.58E-02
de novo miRNA_chr19:1612215..1612261:-	0.00	2.02	-4.95	5.43E-05	2.86E-02
de novo miRNA_chr2:172967274..172967341:-	0.00	2.45	-5.44	7.30E-05	3.36E-02
de novo miRNA_chr2:172967321..172967395:-	0.00	2.45	-5.45	1.08E-04	3.62E-02
de novo miRNA_chr7:51845018..51845089:-	0.00	1.91	-4.80	1.11E-04	3.62E-02
hsa_piR_004307/gb/DQ575881/Homo	3.29	6.24	-3.07	1.18E-04	3.62E-02
Homo_sapiens_chr5.trna22-AspGTC	0.00	2.02	-4.91	1.01E-04	3.62E-02
de novo miRNA_chr4:70136062..70136142:-	0.00	1.55	-4.28	1.52E-04	3.99E-02
de novo miRNA_chr4:70245042..70245122:-	0.00	1.55	-4.28	1.52E-04	3.99E-02

SI 19. Differentially expressed sncRNAs in total plasma samples from early MDS *vs.* advanced MDS. Of the 100 significant sncRNAs (|logFC| > 1 and q < 0.05), only the top 50 genes are listed. logCPM – binary logarithm of counts per million, logFC – binary logarithm of fold change, q-value – FDR adjusted p-value.

gene	logCPM_ early MDS	logCPM_ advanced MDS	logFC	p-value	q-value
hsa-miR-409-5p	6.83	4.99	1.82	1.36E-08	4.86E-05
hsa-miR-1908-5p	8.06	6.43	1.65	6.85E-08	1.19E-04
hsa-miR-3156-5p	2.69	6.31	-3.80	1.34E-07	1.19E-04
Homo_sapiens_chr6.trna152-ValCAC	9.59	7.32	2.27	1.22E-07	1.19E-04
hsa-miR-382-3p	7.51	5.62	1.88	4.62E-07	3.29E-04
hsa-miR-154-5p	6.95	5.33	1.60	1.53E-06	7.77E-04
hsa-miR-181c-5p	6.87	5.46	1.41	1.36E-06	7.77E-04
Homo_sapiens_chr7.trna5-CysGCA	3.66	4.99	-1.40	5.75E-06	2.56E-03
hsa-miR-6852-5p	7.58	5.76	1.82	7.69E-06	2.74E-03
hsa_piR_016658/gb/DQ592931/Homo	13.01	10.86	2.16	7.64E-06	2.74E-03
hsa-miR-103b	12.64	10.65	1.99	1.41E-05	3.42E-03
hsa-miR-221-5p	6.83	5.39	1.44	1.24E-05	3.42E-03
hsa-miR-3692-3p	4.16	5.80	-1.68	1.29E-05	3.42E-03
hsa-miR-494-3p	8.34	6.65	1.69	1.44E-05	3.42E-03
hsa-miR-543	5.81	4.04	1.79	1.21E-05	3.42E-03
de novo miRNA_chr3:4493117..4493175:+	5.69	0.00	8.53	1.83E-05	4.08E-03
hsa-miR-130b-5p	6.29	5.18	1.10	2.08E-05	4.12E-03
de novo miRNA_chr22:32524271..32524358:+	4.33	0.00	7.13	2.00E-05	4.12E-03
hsa-miR-107	12.53	10.86	1.68	2.31E-05	4.33E-03
hsa-miR-374b-5p	7.26	5.72	1.54	3.51E-05	5.46E-03
hsa-miR-4454	8.29	6.64	1.64	3.09E-05	5.46E-03
hsa-miR-98-3p	6.06	5.03	1.02	3.53E-05	5.46E-03
hsa_piR_008112/gb/DQ581031/Homo	14.11	12.42	1.69	3.33E-05	5.46E-03
hsa-miR-374a-5p	8.98	7.15	1.83	4.34E-05	6.42E-03
hsa-miR-6744-5p	5.51	3.34	2.30	4.51E-05	6.42E-03
hsa-miR-1260b	7.97	6.56	1.42	6.21E-05	7.13E-03
hsa-miR-3135b	8.46	7.08	1.38	5.64E-05	7.13E-03
hsa-miR-490-3p	7.06	5.46	1.62	5.46E-05	7.13E-03
hsa-miR-548z	5.29	3.19	2.15	5.54E-05	7.13E-03
hsa-miR-6865-5p	6.93	5.70	1.23	6.02E-05	7.13E-03
U82	3.54	0.00	6.29	5.90E-05	7.13E-03
de novo miRNA_chr3:127478544..127478604:-	3.07	0.00	5.78	6.74E-05	7.43E-03
Homo_sapiens_chr2.trna27-GlyCCC	8.26	7.13	1.13	6.89E-05	7.43E-03
hsa-miR-662	4.45	5.66	-1.27	8.34E-05	8.74E-03
hsa-miR-181d-5p	5.99	4.98	1.03	1.11E-04	1.13E-02
hsa-miR-7853-5p	5.95	3.72	2.28	1.16E-04	1.15E-02

hsa-miR-485-5p	8.62	6.82	1.81	1.20E-04	1.16E-02
hsa-miR-30b-5p	7.30	6.12	1.19	1.54E-04	1.32E-02
hsa-miR-4433a-5p	7.79	5.77	2.03	1.58E-04	1.32E-02
hsa-miR-7-5p	9.80	8.33	1.46	1.55E-04	1.32E-02
de novo miRNA_chr1:228407332..228407406:+	0.00	4.86	-7.68	1.53E-04	1.32E-02
hsa_piR_008113/gb/DQ581032/Homo	14.00	12.55	1.45	1.59E-04	1.32E-02
hsa-miR-766-3p	6.37	5.13	1.26	1.78E-04	1.43E-02
hsa_piR_001169/gb/DQ571525/Homo	2.65	0.00	5.31	1.85E-04	1.43E-02
hsa-miR-302a-5p	2.98	4.32	-1.45	2.16E-04	1.64E-02
hsa-miR-4286	7.90	6.38	1.52	2.26E-04	1.64E-02
hsa-miR-485-3p	10.81	8.93	1.88	2.21E-04	1.64E-02
de novo miRNA_chr12:22183350..22183415:+	0.00	7.32	-10.18	2.35E-04	1.66E-02
Ro-associated	16.01	14.18	1.84	2.38E-04	1.66E-02
Homo_sapiens_chr1.trna74-GluCTC	8.15	6.81	1.35	2.42E-04	1.66E-02

SI 20. Differentially expressed sncRNAs in EV samples from early MDS *vs.* advanced MDS (|logFC| > 1 and q < 0.05). logCPM – binary logarithm of counts per million, logFC – binary logarithm of fold change, q-value – FDR adjusted p-value.

gene	logCPM_ early MDS	logCPM_ advanced MDS	logFC	p-value	q-value
hsa-miR-1260a	6.55	5.34	1.22	1.01E-04	1.92E-02
hsa-miR-1260b	7.30	5.76	1.55	1.31E-05	1.23E-02
hsa-miR-1273c	5.30	4.11	1.23	6.37E-04	4.83E-02
hsa-miR-1299	4.86	6.83	-1.99	3.28E-04	3.08E-02
hsa-miR-181c-3p	5.82	4.62	1.22	1.89E-05	1.23E-02
hsa-miR-1908-5p	6.84	5.68	1.18	1.15E-04	1.92E-02
hsa-miR-197-3p	9.25	8.16	1.10	1.88E-04	2.16E-02
hsa-miR-3151-5p	6.83	5.24	1.61	2.43E-04	2.47E-02
hsa-miR-323b-3p	7.84	6.18	1.68	3.97E-04	3.54E-02
hsa-miR-377-3p	6.94	5.08	1.91	9.64E-05	1.92E-02
hsa-miR-4275	5.30	6.97	-1.70	1.84E-04	2.16E-02
hsa-miR-4286	7.52	5.79	1.76	2.42E-05	1.23E-02
hsa-miR-4307	6.31	4.48	1.87	2.35E-04	2.47E-02
hsa-miR-4433a-5p	7.27	5.07	2.23	3.83E-05	1.52E-02
hsa-miR-4433b-5p	10.15	7.97	2.19	2.38E-04	2.47E-02
hsa-miR-450b-5p	5.61	4.35	1.32	5.07E-05	1.72E-02
hsa-miR-4763-3p	4.60	2.68	2.03	2.38E-05	1.23E-02
hsa-miR-4772-5p	5.79	3.47	2.41	5.30E-05	1.72E-02
hsa-miR-485-3p	9.62	7.67	1.95	1.32E-04	1.92E-02
hsa-miR-5011-3p	2.84	0.07	5.27	1.46E-05	1.23E-02
hsa-miR-625-3p	10.98	9.47	1.51	5.77E-04	4.52E-02
hsa-miR-6754-3p	4.87	3.45	1.46	4.94E-04	4.09E-02
hsa-miR-6787-5p	5.44	6.90	-1.51	6.31E-05	1.74E-02
hsa-miR-6788-3p	4.98	6.55	-1.61	3.04E-06	5.42E-03
hsa-miR-6805-5p	5.86	4.85	1.01	3.17E-05	1.41E-02
hsa-miR-6832-3p	3.89	5.48	-1.66	2.43E-04	2.47E-02
hsa-miR-6857-3p	6.13	7.40	-1.26	8.05E-05	1.79E-02
hsa-miR-6862-5p	5.02	3.68	1.36	3.05E-04	2.94E-02
hsa-miR-6865-5p	6.13	5.08	1.07	5.83E-04	4.52E-02
hsa-miR-7853-5p	7.58	4.46	3.16	1.62E-07	5.76E-04
hsa-miR-7974	3.90	5.28	-1.55	1.37E-04	1.92E-02
de novo miRNA_chr1:27878555..27878644:-	5.47	0.00	8.68	1.42E-04	1.92E-02
de novo miRNA_chr1:33405583..33405644:-	4.00	0.00	7.16	4.38E-04	3.81E-02
de novo miRNA_chr10:129068287..129068327:-	4.94	0.00	8.14	1.46E-04	1.92E-02
de novo miRNA_chr16:57923254..57923331:-	4.04	0.00	7.20	1.32E-04	1.92E-02
de novo miRNA_chr16:72089401..72089448:-	4.59	0.00	7.78	6.78E-05	1.74E-02
de novo miRNA_chr17:58205894..58205949:-	4.75	0.00	7.94	1.27E-04	1.92E-02

de novo miRNA_chr2:202526185..202526242:+	4.88	0.00	8.07	1.34E-04	1.92E-02
de novo miRNA_chr4:185095016..185095060:+	5.25	0.00	8.46	1.55E-04	1.97E-02
hsa_piR_000805/gb/DQ571003/Homo	5.46	3.90	1.63	1.40E-04	1.92E-02
hsa_piR_019420/gb/DQ596670/Homo	5.35	6.74	-1.41	1.80E-04	2.16E-02
hsa_piR_020499/gb/DQ598183/Homo	3.31	4.48	-1.28	5.72E-04	4.52E-02
Homo_sapiens_chr1.trna60-LeuTAA	2.70	0.00	5.73	7.25E-05	1.74E-02

SI 21. Selected miRNAs with different expression levels between early and advanced MDS. Statistical parameters were computed by the exact test in edgeR (*q < 0.05, **q < 0.01, ***q < 0.001). Total plasma - right parts of the graphs, plasma-derived EVs - left parts of the graphs, CTR - controls, MDS1 - early MDS (MDS-SLD, MDS-MLD, and MDS-5q-), MDS2 - advanced MDS (MDS-EB1 and MDS-EB2), AML - AML-MRC.

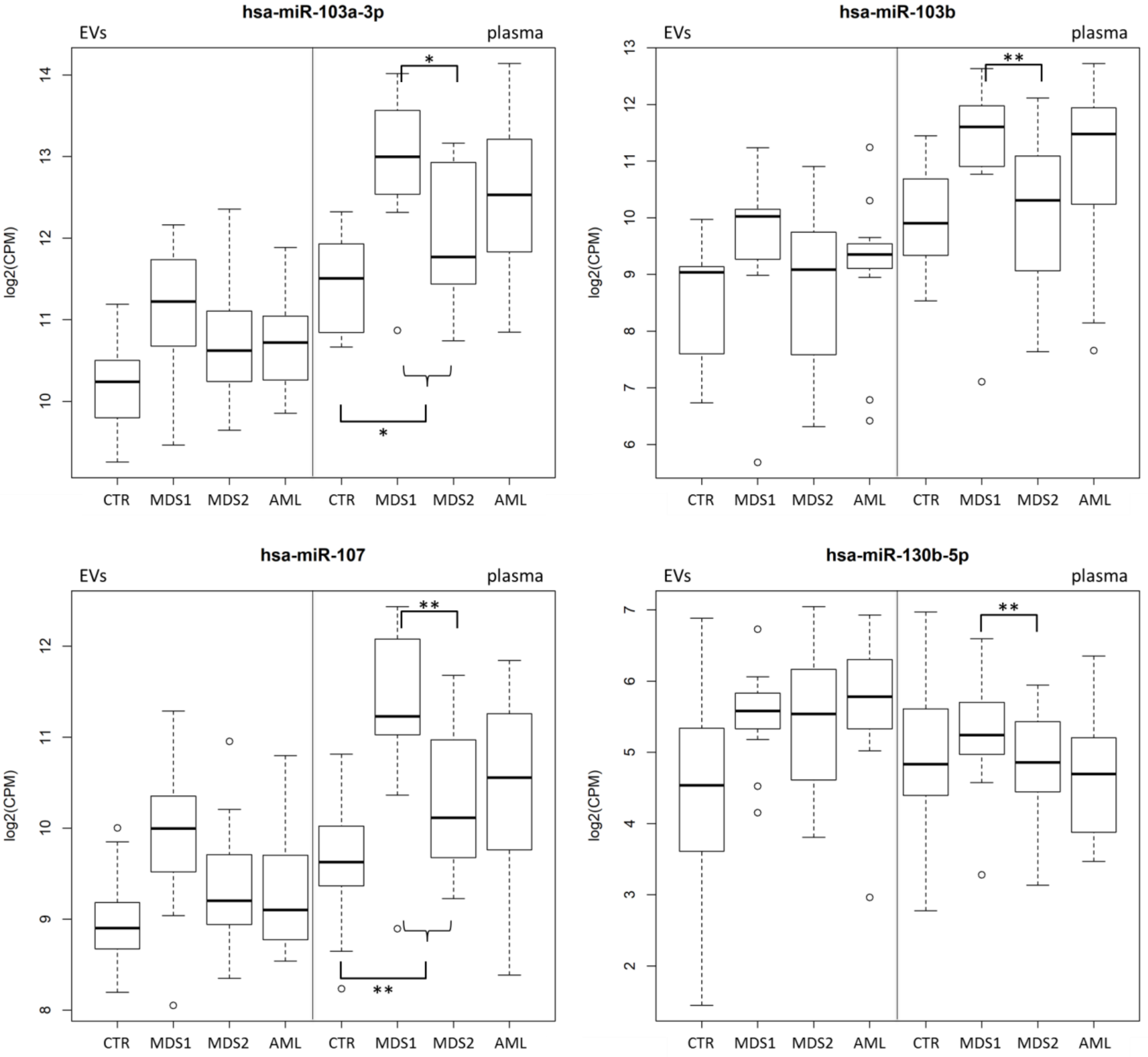

SI 22. miRNAs located within the 14q32 cluster with different expression levels in MDS patients. Statistical parameters were computed by the exact test in edgeR (*q < 0.05, **q < 0.01, ***q < 0.001). Total plasma - right parts of the graphs, plasma-derived EVs - left parts of the graphs, CTR - controls, MDS1 - early MDS (MDS-SLD, MDS-MLD, and MDS-5q-), MDS2 - advanced MDS (MDS-EB1 and MDS-EB2), AML - AML-MRC.

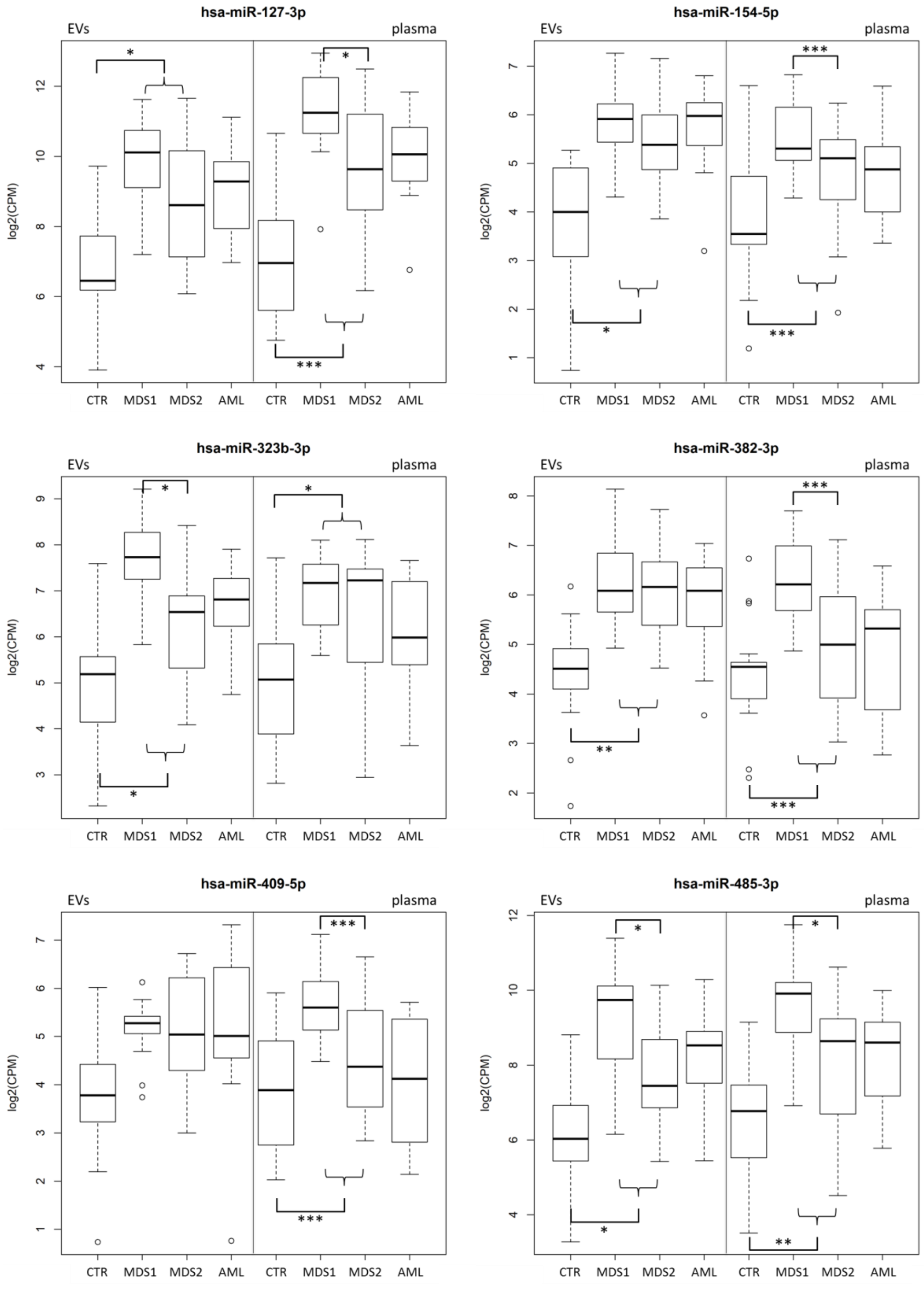

SI 23. Other types of sncRNAs with different expression levels between early and advanced MDS. Statistical parameters were computed by the exact test in edgeR (*q < 0.05, **q < 0.01, ***q < 0.001). Total plasma - right parts of the graphs, plasma-derived EVs - left parts of the graphs, CTR - controls, MDS1 - early MDS (MDS-SLD, MDS-MLD, and MDS-5q-), MDS2 - advanced MDS (MDS-EB1 and MDS-EB2), AML - AML-MRC.

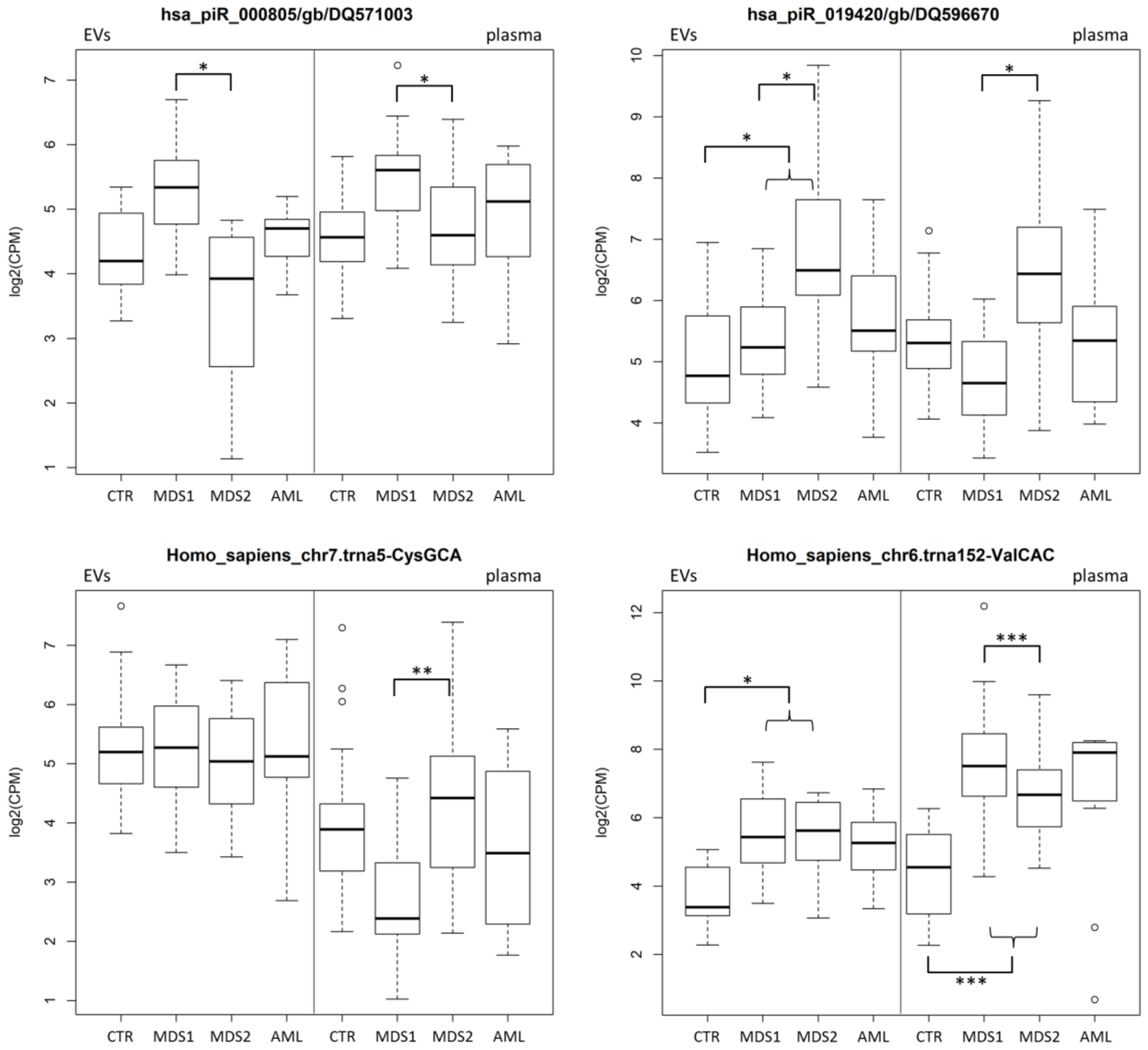

SI 24. Correlation of sncRNA levels measured in the two independent sample cohorts by the two different methods. In the testing cohort, the data were obtained using small RNA-seq (NGS) and in the validation cohort, by the ddPCR method. (A) The mean values of sncRNA levels (miR-16-5p, miR-34a-5p, miR-125a-5p, miR-125b-5p, miR-127-3p, miR-221-3p, and hsa_piR_001170/DQ571526) in individual sample groups (healthy controls, early MDS, advanced MDS, and AML-MRC) were plotted in the graph and Pearson correlation was calculated. (B) Detailed comparison of NGS and ddPCR results for miR-34a levels are shown for illustration.

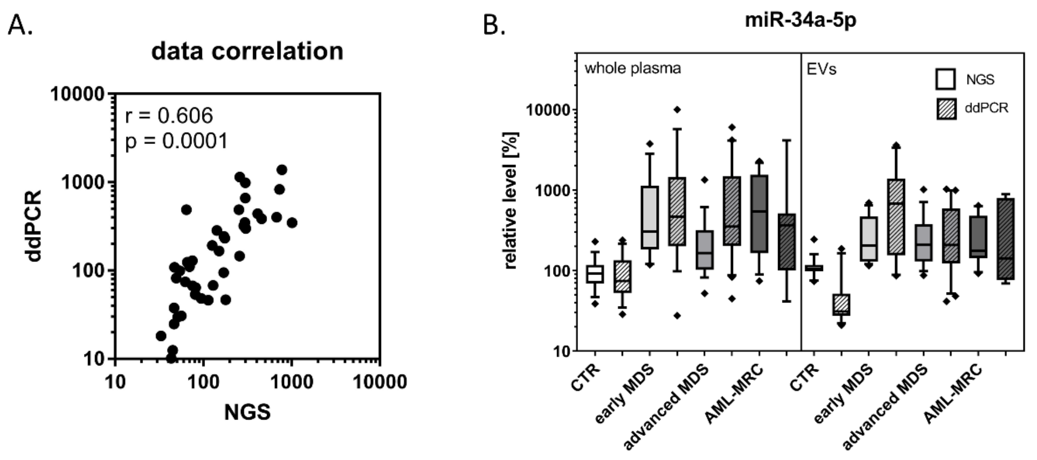

SI 25. Differential sncRNA levels with relation to somatic mutations of (A) the *DNMT3A* gene and (B) the *SF3B1* gene. Statistical parameters were computed by the exact test in edgeR (*p < 0.05, ***p* < 0.01, ***p < 0.001).

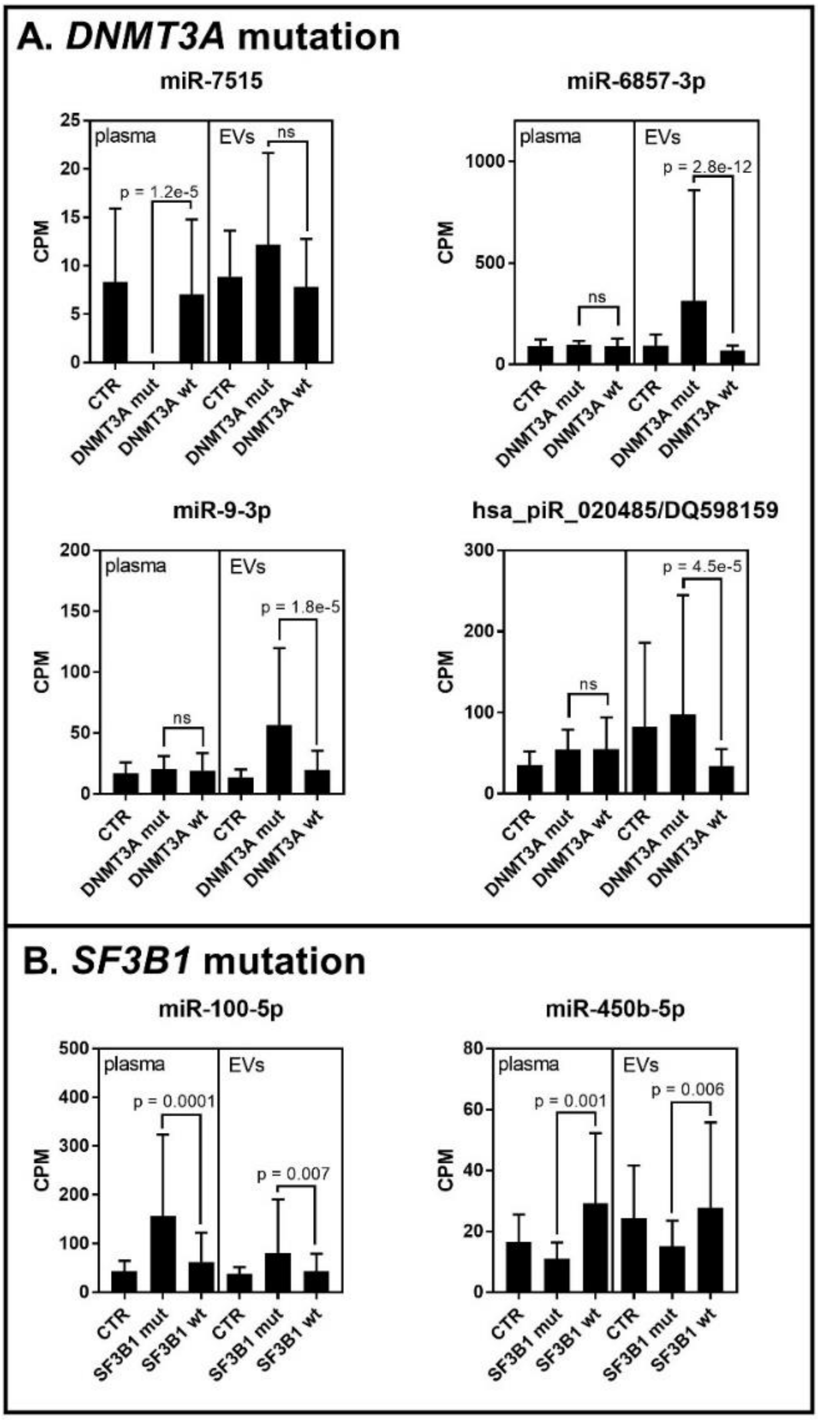

SI 26. SncRNAs significantly ($p < 0.01$) deregulated in MDS patients with *DNMT3A* or *SF3B1* gene mutations.

Somatic mutation		SncRNA category	Upregulated sncRNAs	Downregulated sncRNAs
DNMT3A	total plasma	miRNAs	miR-34b-5p, miR-511-3p, miR-3652, miR-3667-3p, miR-4255, miR-4326, miR-4439, miR-4753-3p, miR-4765, miR-4668-5p, miR-6829-3p, miR-6832-5p	miR-98-3p, miR-133a-3p, miR-135a-5p, miR-340-3p, miR-374a-3p, miR-431-3p, miR-433-3p, miR-505-3p, miR-548ag, miR-708-3p, miR-758-3p, miR-1247-3p, miR-4518, miR-4528, miR-4659b-3p, miR-4781-5p, miR-6842-5p, miR-7515
		piRNAs	-	hsa_piR_016658/DQ592931
		tRNAs	-	chr6.trna120-AlaAGC, chr6.trna87-GluCTC, chr14.trna3-ProTGG
		others	-	-
	plasma-derived EVs	miRNAs	miR-7-5p, miR-9-3p, miR-9-5p, miR-182-5p, miR-183-5p, miR-320c, miR-379-5p, miR-629-5p, miR-1299, miR-3190-5p, miR-4275, miR-4496, miR-4772-5p, miR-5584-3p, miR-6734-5p, miR-6744-5p, miR-6857-3p, miR-6873-3p, miR-7111-3p	miR-513a-5p, miR-887-5p
		piRNAs	hsa_piR_018849/DQ595899, hsa_piR_019420/DQ596670, hsa_piR_019443/DQ596699, hsa_piR_020485/DQ598159	-
		tRNAs	chr11.trna8-SerGCT	-
		others	HBII-85-10, mgU2-19/30, U2, U3-2, U3-2B, U33	-
SF3B1	total plasma	miRNAs	miR-99b-3p, miR-100-5p, miR-1299, miR-3653-5p, miR-4520-5p, miR-4663	miR-424-5p, miR-450b-5p, miR-542-3p, miR-562, miR-767-3p, miR-2052, miR-3692-3p, miR-4646-3p, miR-6744-5p, miR-6812-5p, miR-6828-3p, miR-7161-3p, miR-8054
		piRNAs	hsa_piR_016735/DQ593039	-
		tRNAs	chr1.trna4-GlyCCC	-
		others	5S	-
	plasma-derived EVs	miRNAs	miR-30c-1-3p, miR-100-5p, miR-181b-2-3p, miR-548-3p, miR-597-3p, miR-3155a, miR-6510-5p, miR-6745	miR-450b-5p, miR-744-3p, miR-6857-3p
		piRNAs	-	-
		tRNAs	chr19.trna14-PheGAA	-
		others	U18B, U50	U2

SI 27. A-to-I editing of miRNAs in MDS. (A) Total number of detected A-to-I editing events per sample. (B) Heatmap of the most edited miRNAs. Only the miRNAs edited in ≥ 10 samples with editing ≥ 5% are shown. The black-and-white color scale indicates the level of editing (white – nonedited). (C) Characteristics of the most edited miRNAs. The table summarizes the position of the edited nucleotide in a miRNA, the number of samples with detected editing (out of 118 samples), and the maximal level of detected editing within these samples. (D) Differential editing of miR-411-5p and miR-376c-3p in samples from MDS patients compared to those of healthy controls and AML-MRC patients. eMDS – early MDS, aMDS – advanced MDS, Student's t-test: *p < 0.05, ***p* < 0.01, ***p < 0.001.

A. Total number of editing events **B. The most edited miRNAs**

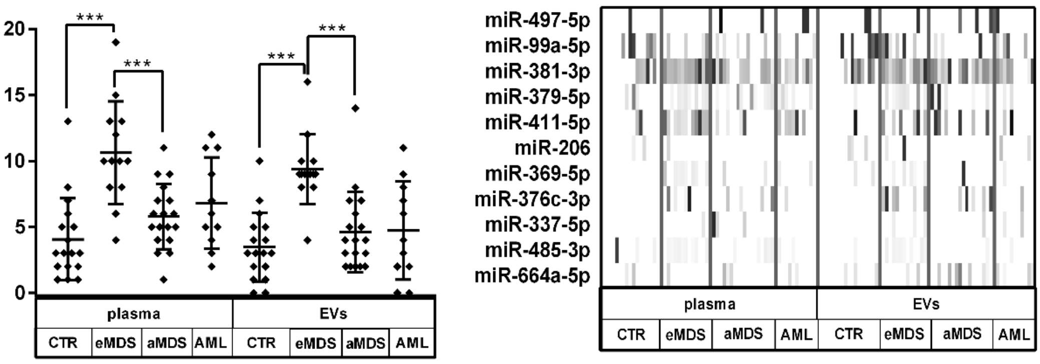

C. Characteristics of the most edited miRNAs

MiRNA	Edited nucleotide	No. of samples with editing	Maximal level of editing
**miR-497-5p**	2	12	42%
**miR-99a-5p**	1	36	36%
**miR-381-3p**	4	79	23%
**miR-379-5p**	5	42	20%
**miR-411-5p**	5	38	17%
**miR-206**	4	10	14%
**miR-369-5p**	3	21	13%
**miR-376c-3p**	6	21	12%
**miR-337-5p**	3	10	8%
**miR-485-3p**	4	30	8%
**miR-664a-5p**	8	24	5%

D. MiRNAs differentially edited in MDS

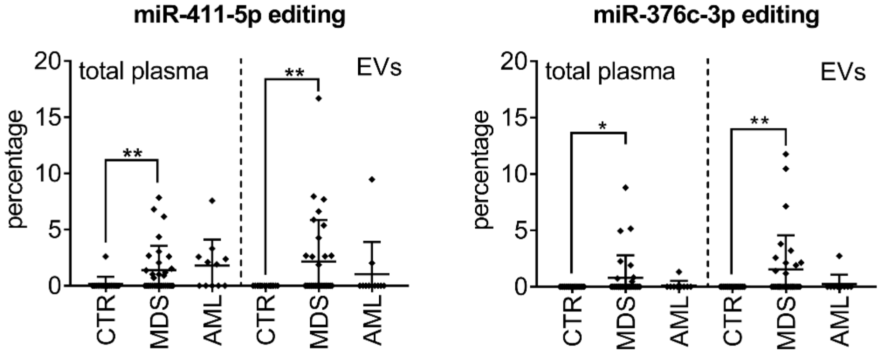

SI 28. List of sncRNAs significantly associated with OS in total plasma (univariate analysis p < 0.05)**.** Only the top 50 genes are listed.

No.	sncRNA	Univariate p-value	Permutation p-value	Hazard Ratio
1	miR-1260b	0.0007295	6e-04	0.441
2	miR-3191-3p	0.000914	9e-04	0.338
3	miR-328-3p	0.000929	8e-04	0.474
4	miR-34c-3p	0.001309	0.0011	6.643
5	miR-4433b-5p	0.0013757	0.001	0.689
6	miR-2277-5p	0.0015207	0.0017	0.245
7	miR-6832-5p	0.0016179	0.0012	3.061
8	miR-1260a	0.001669	9e-04	0.484
9	miR-330-3p	0.0026699	0.0024	0.365
10	miR-1226-3p	0.0031767	0.0036	0.251
11	miR-4433a-3p	0.0031964	0.0023	0.743
12	miR-744-5p	0.0039036	0.0043	0.524
13	miR-6843-3p	0.0040049	0.0045	0.255
14	miR-23c	0.0040442	0.0044	0.487
15	Ro-associated	0.004448	0.0037	0.614
16	miR-4319	0.0045858	0.0029	3.94
17	miR-5047	0.004815	0.0039	3.398
18	miR-4286	0.0053686	0.0053	0.586
19	miR-1273h-3p	0.0056116	0.0056	0.516
20	miR-5583-5p	0.005985	0.0055	2.235
21	miR-671-3p	0.0064734	0.0066	0.542
22	miR-1301-3p	0.0067595	0.0066	0.576
23	miR-511-3p	0.006919	0.0053	1.815
24	miR-4697-3p	0.0069679	0.006	7.18
25	miR-4467	0.006982	0.0095	0.399
26	miR-6852-5p	0.0071556	0.0063	0.619
27	miR-1908-5p	0.0071847	0.0067	0.487
28	miR-429	0.007196	0.0078	0.421
29	ACA36B	0.0073276	0.0077	0.571
30	hsa_piR_020485/gb/DQ598159	0.0074482	0.0069	2.34
31	miR-339-5p	0.0075905	0.0082	0.654
32	miR-6781-5p	0.0077951	0.0072	2.746
33	miR-7-2-3p	0.0082029	0.0076	2.016
34	miR-30d-5p	0.0087168	0.0091	0.506
35	miR-200b-3p	0.0088046	0.0097	0.43
36	miR-4446-3p	0.0091568	0.0079	0.516
37	miR-6844	0.0097256	0.0086	2.971
38	miR-98-3p	0.0098561	0.0097	0.514

No.	sncRNA	Univariate p-value	Permutation p-value	Hazard Ratio
39	miR-139-5p	0.009859	0.0092	0.501
40	miR-205-3p	0.0101743	0.0095	1.902
41	miR-6814-3p	0.0115089	0.0127	2.858
42	miR-28-3p	0.0116952	0.0132	0.637
43	miR-628-3p	0.0117599	0.0142	0.5
44	miR-151b/151a-5p	0.0120327	0.0139	0.609
45	miR-4740-3p	0.012111	0.0131	3.038
46	miR-4423-5p	0.0121473	0.0123	2.287
47	miR-7974	0.0121517	0.0156	1.858
48	chr6.trna126-LeuAAG	0.0123017	0.0126	0.569
49	miR-766-3p	0.0123959	0.0132	0.433
50	miR-625-5p	0.0126746	0.0132	0.522

SI 29. List of sncRNAs significantly associated with OS in the EV fraction (univariate analysis p < 0.05)**.** Only the top 50 genes are listed.

No.	sncRNA	Univariate p-value	Permutation p-value	Hazard Ratio
1	miR-1237-3p	1.73e-05	< 1e-07	20.135
2	U33	0.0005899	6e-04	2.499
3	miR-548av-5p/548k	0.0012609	0.001	0.217
4	hsa_piR_019420/gb/DQ596670	0.0013097	0.001	2.235
5	miR-4799-5p	0.0028959	0.0011	0.153
6	miR-424-3p	0.00313	0.005	4.886
7	miR-5705	0.0042035	0.0052	3.125
8	miR-5707	0.0042469	0.0025	0.319
9	miR-148a-5p	0.0044003	0.0046	0.199
10	miR-4689	0.0054431	0.0059	3.015
11	miR-4474-3p	0.005792	0.0085	3.55
12	miR-3611	0.0062224	0.0042	0.363
13	miR-4433b-5p	0.0065335	0.0061	0.685
14	miR-3945	0.0072613	0.0085	3.082
15	U3-2B	0.0088581	0.0084	1.613
16	miR-6872-3p	0.0102911	0.0094	0.429
17	miR-767-5p	0.0103127	0.0088	0.483
18	miR-181c-5p	0.0108319	0.0166	0.479
19	miR-3145-3p	0.0108857	0.013	0.485
20	miR-6780b-3p	0.0110434	0.0159	2.959
21	HBII-85-10	0.0125239	0.01	1.997
22	miR-4446-3p	0.0127126	0.0126	0.515
23	miR-548ar-5p	0.0128565	0.0112	0.251
24	chr11.trna8-SerGCT	0.0129431	0.012	2.1
25	miR-6840-3p	0.0145525	0.0143	0.458
26	Ro-associated	0.0146849	0.0144	0.637
27	chr6.trna65-AlaAGC	0.0156566	0.0198	0.392
28	miR-3145-5p	0.0157297	0.0125	0.403
29	miR-1263	0.0158345	0.0146	0.395
30	miR-6767-5p	0.016836	0.0143	1.719
31	miR-1306-3p	0.0168833	0.0175	0.289
32	hsa_piR_020365/gb/DQ597975	0.0169372	0.0188	1.745
33	U3-2	0.0180341	0.0154	1.549
34	miR-1273c	0.0185835	0.0201	0.607
35	miR-3674	0.0188046	0.0182	0.5
36	U3	0.0189568	0.0214	1.536
37	miR-6808-3p	0.0191047	0.0235	0.384
38	miR-200b-3p	0.0192423	0.0183	0.357

No.	sncRNA	Univariate p-value	Permutation p-value	Hazard Ratio
39	miR-6078	0.0199924	0.0202	2.401
40	miR-939-5p	0.020072	0.0196	3.875
41	miR-1282	0.0207642	0.0221	2.525
42	let-7b-3p	0.0238514	0.027	1.891
43	miR-6837-5p	0.0255722	0.0274	2.077
44	miR-8080	0.0257791	0.0229	0.428
45	miR-2276-5p	0.025936	0.0279	0.313
46	miR-580-5p	0.0260879	0.0266	0.57
47	miR-206	0.0265768	0.0298	1.602
48	miR-4798-5p	0.0266794	0.0255	0.565
49	miR-4433b-3p	0.0275745	0.0267	0.478
50	U3-4	0.0288776	0.031	1.553

SI 30. Kaplan-Meier curves for individual sncRNAs significantly associated with overall survival of MDS patients in samples of (A) total plasma and (B) plasma-derived EVs.

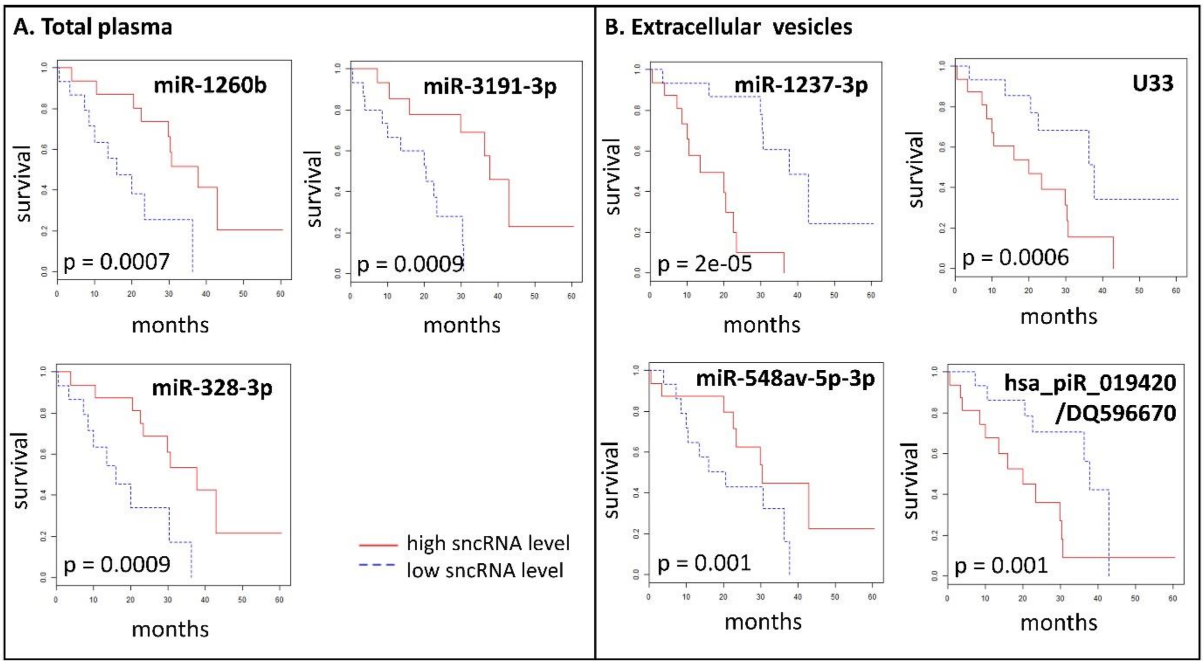

SI 31. Correlation of clinical variables with individual sncRNA levels and with combined scores for OS. The Pearson correlation coefficient is listed. *** $p < 0.001$, ** $p < 0.01$, * $p < 0.05$.

		age	blasts	hemoglobin	neutrophils	platelets	karyotype
Total plasma	PL miR-1260b	-0.334	-0.510**	0.046	0.015	0.838***	-0.225
	PL miR-3191-3p	-0.253	-0.199	-0.125	0.061	0.337	-0.300
	PL miR-328-3p	-0.453*	-0.487**	0.239	0.029	0.826***	-0.203
	combined score (total plasma)	0.284	0.531**	-0.088	-0.053	-0.818***	0.218
EVs	EV U33	0.090	0.365*	-0.229	0.146	-0.372*	-0.093
	EV hsa_piR_019420/DQ596670	0.090	0.412*	-0.119	0.140	-0.473**	-0.053
	EV miR-548av-5p	0.045	-0.207	0.357*	-0.114	0.270	-0.204
	combined score (EVs)	0.168	0.413*	-0.374*	0.066	-0.490**	0.264

SI 32. Circulating sncRNAs deregulated in pretreatment AZA patients with respect to their response to the treatment according to differential expression analysis (|logFC| > 1, q < 0.05).

SncRNAs (logFC; q)	Total plasma		EV fraction	
	**responders vs. progressors**	**responders vs. nonresponders**	**responders vs. progressors**	**responders vs. nonresponders**
**Upregulation in responders**	miR-4774-3p (5,61; 0.033)	miR-762 (1.35; 0.039)	miR-6857-3p (1.64; 0.024)	miR-6857-3p (1.72; 0.00078)
				miR-1299 (2.51; 0.040)
				miR-183-5p (2.59; 0.040)
**Downregulation in responders**	miR-125b-5p (-4.22; 1.23E-09)	miR-125b-5p (-3.59; 0.0015)	miR-513b-3p (-3.49; 0.0017)	miR-513b-3p (-2.89; 0.017)
	miR-4324 (-2.31; 0.023)	miR-3156-5p (-3.39; 0.039)	miR-4275 (-2.53; 0.028)	miR-6832-3p (-2.21; 0.019)
		miR-3692-3p (-1.77; 0.039)		

SI 33. SUPPLEMENTARY METHODS

Patients

The main study cohort included peripheral blood (PB) plasma samples from 31 MDS and 11 AML-MRC patients analyzed by small RNA-seq. An independent validation cohort included 36 MDS and 7 AML-MRC patients analyzed by droplet digital PCR (ddPCR). The PB samples were obtained from patients during routine clinical assessment at the Institute of Hematology and Blood Transfusion and the First Department of Internal Medicine, General Faculty Hospital, Prague. Samples were collected from patients with primary MDS with no known history of previous malignancy, chemotherapy or radiation therapy. None of the patients had received drug therapy for their disease or hematopoietic stem cell transplantation (HSCT) prior to blood collection. After sample collection, several MDS/AML patients received azacytidine (AZA) therapy. AZA was administered at 75 mg/m^2/day for 7 consecutive days every 28 days. The hematological evaluation of the response to the treatment was performed after cycle 4 according to the International Working Group (IWG) criteria for MDS [1] and AML [2]. As controls, blood plasma samples from 29 age-matched healthy donors (17 individuals analyzed by small RNA-seq and 12 individuals by ddPCR) with no adverse medical history were used. Written informed consent was obtained from all tested subjects in accordance with the approval from the Institutional Review Board.

Separation of blood plasma

PB was collected in EDTA tubes, and plasma was separated from the blood by centrifugation at 460 *g* for 10 min. The absence of hemolysis in plasma was confirmed spectrophotometrically by measuring the free hemoglobin (absorbance at 414 nm) and by evaluating the difference between miR-451 and miR-23a levels by qPCR using the StepOnePlus Real-Time PCR System (Thermo Fisher Scientific, Waltham, MA, USA) as described previously [3]. Plasma samples were further centrifuged at 12000 *g* at 4°C for 15 min to remove cell debris and stored at -80°C.

TEM, NTA, and western blotting performance

To confirm the presence and size of EVs, transmission electron microscopy (TEM), nanoparticle tracking analysis (NTA), and western blotting were performed. For TEM, EVs were visualized by negative staining. Parlodion-carbon-coated grids were floated on the top of a 5 µl drop of the sample for 5 min. Then, the grids were transferred on the top of a drop of 2% phosphotungstic acid (pH 7.4), stained for 2×1 min and dried. Photomicrographs were taken with a JEOL JEM-1011 electron microscope (JEOL, Peabody, MA, USA) operated at 80 kV. NTA was performed using a Malvern NanoSight NS300 instrument (Malvern Panalytical, Malvern, UK). Briefly, purified EVs were diluted 5 x 10^3 in PBS and tracked using NTA analysis software. Each sample was analyzed 3 times, and the counts were merged.

For western blotting, EVs and K562 total cell lysate (used as a positive or a negative control) were lysed in 200 µl of NaCl-HEPES + 0,15% Triton (Sigma-Aldrich, St. Louis, MO, USA) and incubated on ice for 20 min. Total protein concentration was quantified by the Bradford

protein assay (Bio-Rad, Hercules, CA, USA) and 30 µg of proteins were separated on 4 - 15% Mini-PROTEAN TGXTM gels (Bio-Rad) and transferred to a polyvinylidene difluoride (PVDF) membrane. The membrane was blocked with SuperBlock blocking buffer (Thermo Fisher Scientific, Waltham, MA, USA) and immunostained. The following primary antibodies were used: mouse anti-CD81 antibody (1:1000) (B11, sc-166029, Santa Cruz Biotechnology, Dallas, Texas, USA), rabbit anti-CD9 antibody (1:1000) (EXOAB Kit 1, System Biosciences, Palo Alto, CA, USA) and rabbit anti-calnexin (C5C9) antibody (1:1000) (2679, Cell Signaling Technology, Danvers, MA, USA). Secondary horseradish peroxidase-conjugated anti-rabbit (7074P2, Cell Signaling Technology) or anti-mouse (7076P2, Cell Signaling Technology) antibodies were used.

RNA extraction

RNA was extracted from total plasma (200 µL) and from isolated EV fractions using the miRNeasy Serum/Plasma Kit (QIAGEN, Hilden, Germany) according to the manufacturer's protocol. The concentration of RNA was quantified with a Qubit 2.0 fluorometer (Thermo Fisher Scientific).

Small RNA-seq and data analysis

All sequencing libraries were constructed from 5 µl of extracted plasma RNA and extravesicular RNA. The libraries were prepared, amplified and purified using a QIAseq miRNA Library Kit (QIAGEN) following the manufacturer's protocol. The concentration and size of the purified libraries were measured by Qubit 2.0 fluorometer and Agilent 4200 TapeStation (Agilent, Santa Clara, CA, USA), respectively. All libraries were adjusted to 4 nM, pooled together, and sequenced on a HiSeq 2500 sequencer (Illumina, San Diego, CA, USA) as single reads for 83 cycles.

After quality control of raw data using the FastQC tool [4], the sequences were processed using the QIAseq miRNA Primary Quantification pipeline (QIAGEN). Briefly, 3' adapter and low-quality bases were trimmed using Cutadapt [5], and the insert sequences and unique molecular indices (barcodes) were identified. Reads shorter than 16 nucleotides were discarded from the analysis. Sequences were then aligned using a sequential alignment strategy to map to different databases (perfect match to miRBase mature, miRbase hairpin, noncoding RNA, mRNA and other RNA, and finally a second mapping to miRBase mature, where up to two mismatches were tolerated) using Bowtie [6]. At each step, only unmapped sequences were passed to the next step. miRBase V21 was used for annotation of miRNAs, and piRNABank was used for piRNAs. All remaining unaligned sequences were mapped to the GRCh38 genome. *De novo* miRNAs were predicted using the miRdeep2 tool. Annotated read counts were subsequently processed in R statistical environment. Data normalization and subsequent statistical analyses were performed using the edgeR package [7]. Binary logarithms of fold changes (logFC) and q-values (False Discovery Rate (FDR) adjusted p-value) were generated as an output of edgeR package for differential expression analysis of the data. Hierarchical cluster analysis was performed using the pvclust package [8] with average

correlation. The remaining reads unmapped to the human genome were analyzed by the metagenome analyzer MEGAN [9] on some of the samples (11 patients and 3 healthy controls) using 50,000 randomly selected reads (the number was assessed as sufficient for the analysis based on a taxonomy rarefaction plot). Analysis of A-to-I editing was performed using the miRge2.0 tool [10].

Mutational screening and data analysis

Mutational screening was performed as a part of routine clinical assessment using the TruSight Myeloid Sequencing Panel Kit (Illumina) containing 568 amplicons of 54 genes associated with myeloid malignancies. The amplicon library was constructed according to the manufacturer's recommendations. The libraries were 2x150 bp paired-end sequenced on a MiSeq instrument (Illumina), and the data were analyzed using NextGENe software (SoftGenetics, State College, PA, USA). The clinical significance of each variant was verified in several genomic databases (UCSC, COSMIC, ExAC, and PubMed). The arbitrary cut off was set at 3% of variant allele frequency (VAF).

Droplet digital PCR (ddPCR)

The quantity of individual miRNAs was verified by ddPCR using a QX200 ddPCR system (Bio-Rad, Hercules, CA, USA). First, miRNAs were reverse-transcribed by a TaqMan MicroRNA Reverse Transcription Kit (Thermo Fisher Scientific) following the manufacturer´s instructions. PCR reactions were prepared with ddPCR Supermix for Probes (Bio-Rad) and TaqMan miRNA assays (Thermo Fisher Scientific). Droplets were prepared using QX200 Automated Droplet Generator and (after PCR) counted on QX200 Droplet Reader with QuantaSoft software (all from Bio-Rad).

Statistical analyses

Statistical analyses were performed using GraphPad Prism v7 software (La Jolla, CA, USA). Student's t-tests were used to compare continuous variables between different groups of samples. Correlation analyses were performed by computing Pearson correlation coefficients (r). Differences were considered statistically significant at $p < 0.05$.

The overall survival (OS)-associated sncRNAs were identified by performing univariate Cox regression along with a permutation test using BRB-ArrayTools [11]. sncRNAs with permutation p-values < 0.001, which were computed based on 10,000 random permutations, were considered significantly associated with survival and included in a combined sncRNA signature. Then, a formula of survival risk score was constructed by including each of the selected sncRNAs, weighted by their estimated regression coefficients in the univariate Cox regression model. The leave-one-out cross-validation (LOOCV) method was employed to evaluate the accuracy of the score system. The prognostic index of a sample was computed by the formula $\sum_i w_i x_i + 6.861$, where w_i was the estimated regression coefficient and x_i was the logged level for the *i*-th gene. The patient cohort was then partitioned into two risk groups according to the survival risk score (>0 for the high-risk group and ≤0 for the low-risk group).

Then, Kaplan–Meier curves and receiver operating characteristic (ROC) curves were plotted, and area under curve (AUC) values were calculated. Multivariate Cox regression analysis was performed to identify independent variables associated with patient survival.

The Recursive Feature Elimination (RFE) method implemented in Support Vector Machine (SVM) regression model was used to define sncRNA classifiers that discriminated AZA responders and nonresponders based on sncRNA pretreatment levels and interactions between them. Accuracy (Acc) and AUC were calculated to define the optimal number of features. Since the data sample was limited, the LOOCV method was employed to provide an unbiased evaluation of a model fit. The group of best classifiers was further tested and reduced by backward stepwise logistic regression algorithms using the maximum likelihood estimation (MLE) method. The resulting predictive formula was calculated by logistic regression using the KNIME platform and sag solver.

Pathway analysis

Pathway analysis was performed based on significant differences in miRNA levels using DIANA-miRPath v3.0 [12]. Within the analysis, target prediction of miRNAs was computed using the DIANAmicroT-CDS, and the most significantly affected KEGG pathways were identified.

SUPPLEMENTARY REFERENCES

[1] Cheson BD, Greenberg PL, Bennett JM, Lowenberg B, Wijermans PW, Nimer SD et al. Clinical application and proposal for modification of the International Working Group (IWG) response criteria in myelodysplasia. Blood 2006;108:419-25.

[2] Cheson BD, Bennett JM, Kopecky KJ, Büchner T, Willman CL, Estey EH et al. Revised recommendations of the International Working Group for Diagnosis, Standardization of Response Criteria, Treatment Outcomes, and Reporting Standards for Therapeutic Trials in Acute Myeloid Leukemia. J Clin Oncol 2003;21:4642-9.

[3] Jaynish S Shah, Patsy S Soon, Deborah J Marsh. Comparison of Methodologies to Detect Low Levels of Hemolysis in Serum for Accurate Assessment of Serum microRNAs. PLoS One 2016;11:e0153200.

[4] Wingett SW, Andrews S. FastQ Screen: A tool for multi-genome mapping and quality control. F1000Res 2018;7:1338.

[5] Martin M. Cutadapt removes adapter sequences from high-throughput sequencing reads. EMBnet.journal 2011.

[6] Langmead B, Trapnell C, Pop M, Salzberg SL. Ultrafast and memory-efficient alignment of short DNA sequences to the human genome. Genome Biol 2009;10:R25.

[7] Robinson MD, McCarthy DJ, Smyth GK. edgeR: a Bioconductor package for differential expression analysis of digital gene expression data. Bioinformatics 2010;26:139-40.

[8] Suzuki R, Shimodaira H. Pvclust: an R package for assessing the uncertainty in hierarchical clustering. Bioinformatics 2006;22:1540-2.

[9] Huson DH, Beier S, Flade I, Górska A, El-Hadidi M, Mitra S et al. MEGAN Community Edition - Interactive Exploration and Analysis of Large-Scale Microbiome Sequencing Data. PLoS Comput Biol 2016;12:e1004957.

[10] Lu Y, Baras AS, Halushka MK. miRge 2.0 for comprehensive analysis of microRNA sequencing data. BMC Bioinformatics 2018;19:275.

[11] Ishwaran H, Kogalur UB, Gorodeski EZ, Minn AJ, Lauer MS. High-dimensional variable selection for survival data. J AM STAT ASSOC 2010;105:205-17.

[12] Vlachos IS, Zagganas K, Paraskevopoulou MD, Georgakilas G, Karagkouni D, Vergoulis T et al. DIANA-miRPath v3.0: deciphering microRNA function with experimental support. Nucleic acids research 2015;43:W460-6.
